# Approximating non linear higher order ODEs by a three point block algorithm

**DOI:** 10.1371/journal.pone.0246904

**Published:** 2021-02-12

**Authors:** Ahmad Fadly Nurullah Rasedee, Mohammad Hasan Abdul Sathar, Khairil Iskandar Othman, Siti Raihana Hamzah, Norizarina Ishak

**Affiliations:** 1 Fakulti Ekonomi dan Muamalat, Universiti Sains Islam Malaysia, Nilai, Negeri Sembilan, Malaysia; 2 Centre of Foundation Studies for Agricultural Science, Putra University of Malaysia, Serdang, Selangor, Malaysia; 3 Faculty of Computer and Mathematical Sciences, Universiti Teknologi MARA, Shah Alam, Selangor Darul Ehsan, Malaysia; 4 Fakulti Sains dan Teknologi, Universiti Sains Islam Malaysia, Nilai, Negeri Sembilan, Malaysia; Central University of Karnataka, INDIA

## Abstract

Differential equations are commonly used to model various types of real life applications. The complexity of these models may often hinder the ability to acquire an analytical solution. To overcome this drawback, numerical methods were introduced to approximate the solutions. Initially when developing a numerical algorithm, researchers focused on the key aspect which is accuracy of the method. As numerical methods becomes more and more robust, accuracy alone is not sufficient hence begins the pursuit of efficiency which warrants the need for reducing computational cost. The current research proposes a numerical algorithm for solving initial value higher order ordinary differential equations (ODEs). The proposed algorithm is derived as a three point block multistep method, developed in an Adams type formulae (3PBCS) and will be used to solve various types of ODEs and systems of ODEs. Type of ODEs that are selected varies from linear to nonlinear, artificial and real life problems. Results will illustrate the accuracy and efficiency of the proposed three point block method. Order, stability and convergence of the method are also presented in the study.

## Introduction

The multistep method was discovered by Bashforth and Adams [1] in their pursue to extend Euler’s method. The idea which was then named the Adams-Bashforth method was formulated by obtaining the approximated solution for a point by way of solution values from multiple previous steps. Milne [2, 3] then established a new form of multistep method, known as the predictor-corrector formulation. The modern multistep method was widely researched by authors such as [4–9].

Krogh [6], managed to revitalize the field of numerical method for solving ordinary differential equations (ODEs) which was almost discarded as robust, with a divided difference approach. The premise of his work is that the back values of any point of the derivative could be interpolated. In the same study, Krogh presented a comparison for two second order problems similar to the problem provide in [5], where Gear had introduced a Nordsieck version of the multi step method.

The current study is part of research series motivated by [9]. Suleiman [9] developed an algorithm for solving stiff and non stiff higher order ordinary differential equations. The nonstiff portion of the algorithm was derived in a divided difference formulation whereas the stiff algorithm was red obtained using a backward differentiation formulation. Suleiman [9] introduced a divided difference formulation known as Direct Integration (DI) method for solving nonstiff higher order ODEs directly. Omar [10] then established a new variation of the DI method by implementing a block algorithm. The block DI method developed by Omar consists of two different red algorithms, namely explicit block method and implicit block method algorithms. The block method developed in Omar [10] was matched by a fully implicit block method established by Abdul Majid [11]. In [12], authors presented a predictor-corrector algorithm in backward difference form for solving higher ODEs. The backward difference formulation from [12] was then extended in [13]. Md Ijam [13] fitted the backward difference formulae with a two point block algorithm, reducing the number of function evaluations by half.

The current study is established to overcome drawbacks of the DI method as well to provide an efficient numerical multistep method. When establishing a numerical method, accuracy has constantly been the benchmark to determine its viability. But, as numerical methods become more and more robust, accuracy alone is longer sufficient hence, the need for efficiency. The efficiency of a numerical method is associated with computational cost which translates into accuracy per step. The current study proposes a three-point block multistep formulation (3PBCS) in backward difference form for solving higher order ODEs directly. The three-point formulation requires only a fraction of computational steps usually required by standard methods. Complemented with an Adams equivalent predictor-corrector algorithm, the 3PBCS method is able to reduce computational cost more significantly. The drawback of the 3PBCS method, is the need for establishing three set of coefficients for each blocks but, by establishing multiple recursive relationships between explicit and implicit coefficients and also for coefficients of different orders, the corrector algorithm can be written in terms of the predictor which eliminates redundancy in the form of unnecessary calculations. This issue becomes irrelevant if introduced with a parallel algorithm.

Recent advancement in related area can be found in works such as [14–27] and others.

## Establishing the three-point block method

Ordinary differential equations are often used to model the occurrence of natural phenomenon to man made mechanics. Among these applications include modeling two body motions, chemical reactions and engineering problems such as the bend of a thin clamped beam. These ODE models can be categorized into stiff and nonstiff. However, the study conducted here focuses mainly on initial value nonstiff ODEs of any order. We begin by considering the higher order ODE,

$$\begin{array}{c} f^{\left(n\right)}=\phi (t,f,f^{\prime },\ldots ,f^{\left(n-1\right)}) \end{array}$$ (1)

with the initial conditions

$$\begin{array}{c} f(\alpha )=\phi_{0},\;f^{\prime }(\alpha )=\phi_{0}^{\prime },\;f^{\prime \prime }(\alpha )=\phi_{0}^{\prime \prime },\;\ldots \ldots \;,\;f^{\left(n-1\right)}(\alpha )=\phi_{0}^{\left(n-1\right)}. \end{array}$$

In a three point block formulation, derivation of all three points are necessary. But, to avoid unwanted redundancy, only derivation of the third point will be elaborated. Comprehensive derivation of the first and second points can be obtained from [28] and [13]. When formulating a predictor-corrector three-point block algorithm, obtaining the explicit and implicit integration coefficients is crucial. The proposed three-point block method follows similar formulation to the Adam-Basthforth-Moulton method.

### The predictor

The third point predictor is derived by integrating (1) once with the limit of integration from *t*_*i*_ to *t*_*i*+3_ which is then denoted as

$$\begin{array}{c} f_{p}^{\left(n-1\right)}\left(t_{i+3}\right)=f_{p}^{\left(n-1\right)}\left(t_{i}\right)+\int_{t_{i}}^{t_{i+3}}\phi (t,f,f^{\prime },\ldots ,f^{\left(n-1\right)})dt. \end{array}$$ (2)

Then, we approximate *ϕ*(*t*, *f*, *f*′, …, *f*^(*n*−1)^) using the Newton Gregory backward difference polynomial

$$\begin{array}{c} P_{i}(t)=\sum_{j=0}^{k-1}\left(-1{)}^{j}(\begin{array}{c} -s\\ j \end{array}\right)\nabla^{j}\phi_{i},\;s=\frac{t-t_{i+3}}{h}, \end{array}$$

and substituting *dt* = *hds*, thus allowing Eq (2) to be rewritten as

$$\begin{array}{c} f_{p}^{\left(n-1\right)}\left(t_{i+3}\right)=f_{p}^{\left(n-1\right)}\left(t_{i}\right)+h\int_{0}^{3}\sum_{j=0}^{k-1}\left(-1{)}^{j}(\begin{array}{c} -s\\ j \end{array}\right)\nabla^{j}\phi_{i}ds. \end{array}$$ (3)

Next, the integral in (3) is substituted by

$$\begin{array}{c} \beta_{3,1,j}^{p}=(-1{)}^{j}\int_{0}^{3}(\begin{array}{c} -s\\ j \end{array})ds, \end{array}$$ (4)

which provides the following first order predictor formulation

$$\begin{array}{c} f_{p}^{\left(n-1\right)}\left(t_{i+3}\right)=f_{p}^{\left(n-1\right)}\left(t_{i}\right)+h\sum_{j=0}^{k-1}\beta_{3,1,j}^{p}\nabla^{j}\phi_{i}. \end{array}$$

This is then followed by derivation of the second order predictor formulation. The second order predictor is established by integrating (1) twice, which results to the following

$$\begin{array}{c} f_{p}^{\left(n-2\right)}\left(t_{i+3}\right)=f_{p}^{\left(n-2\right)}\left(t_{i}\right)+hf_{p}^{\left(n-1\right)}\left(t_{i}\right)+h\int_{t_{i}}^{t_{i+3}}(t_{i+3}-t)\phi (t,f,f^{\prime },\ldots ,f^{\left(n-1\right)})\;dt. \end{array}$$

Again, we substitute *ϕ*(*t*, *f*, *f*′, …, *f*^(*n*−1)^) with the Newton Gregory backward difference polynomial then replacing (*t* − *t*_*i*+3_) by *h*(3 − *s*) where *s* is as previously defined, yields

$$\begin{array}{c} f_{p}^{\left(n-2\right)}\left(t_{i+3}\right)=f_{p}^{\left(n-2\right)}\left(t_{i}\right)+hf_{p}^{\left(n-1\right)}\left(t_{i}\right)+h^{2}\int_{0}^{3}\left(3-s\right)\sum_{j=0}^{k-1}\left(-1{)}^{j}(\begin{array}{c} -s\\ j \end{array}\right)\nabla^{j}\phi_{i}\;ds. \end{array}$$

Then, replacing the integral

$$\begin{array}{c} \beta_{3,2,j}^{p}=(-1{)}^{j}\int_{0}^{3}\left(3-s\right)(\begin{array}{c} -s\\ j \end{array})ds, \end{array}$$ (5)

gives

$$\begin{array}{c} f_{p}^{\left(n-2\right)}\left(t_{i+3}\right)=f_{p}^{\left(n-2\right)}\left(t_{i}\right)+hf_{p}^{\left(n-1\right)}\left(t_{i}\right)+h^{2}\sum_{j=0}^{k-1}\beta_{3,2,j}^{p}t^{j}\nabla^{j}\phi_{i}. \end{array}$$

For the *n*^*th*^ order integration, (1) is integrated *n* fold,

$$\begin{array}{c} \begin{array}{cc} f_{p}\left(t_{i+3}\right)=f_{p}\left(t_{i}\right) & +hf_{(}^{\prime }t_{i})+\ldots +\frac{h^{\left(n-1\right)}}{(n-1)!}f_{p}^{\left(n-1\right)}\left(t_{i}\right)\\ & +h\int_{t_{i}}^{t_{i+3}}\frac{{\left(t_{i+3}-t\right)}^{\left(n-1\right)}}{(n-1)!}\phi (t,f,f^{\prime },\ldots ,f^{\left(n-1\right)})\;dt. \end{array} \end{array}$$

Implementing similar step as the previous order, yields

$$\begin{array}{c} f_{p}\left(t_{i+3}\right)=f_{p}\left(t_{i}\right)+hf_{p}^{\prime }\left(t_{i}\right)+\ldots +\frac{h^{\left(n-1\right)}}{(n-1)!}f_{p}^{\left(n-1\right)}\left(t_{i}\right)+h^{\left(n-1\right)}\sum_{j=0}^{k-1}\beta_{3,n,j}^{p}t^{j}\nabla^{j}\phi_{i}. \end{array}$$ (6)

where

$$\begin{array}{c} \beta_{3,d,j}^{p}=(-1{)}^{j}\int_{0}^{3}\frac{{\left(3-s\right)}^{\left(n-1\right)}}{(n-1)!}(\begin{array}{c} -s\\ j \end{array})ds. \end{array}$$

### The corrector

Derivation of the corrector also begins with integrating (1) once, which introduces the corrector as follows

$$\begin{array}{c} f_{c}^{\left(n-1\right)}\left(t_{i+3}\right)=f_{c}^{\left(n-1\right)}\left(t_{i}\right)+\int_{t_{i}}^{t_{i+3}}\phi (t,f,f^{\prime },\ldots ,f^{\left(n-1\right)})dt. \end{array}$$

Again, approximating *ϕ*(*t*, *f*, *f*′, …, *f*^(*n*−1)^) using the Newton Gregory backward difference polynomial with some subtle differences,

$$\begin{array}{c} P_{n+3}(t)=\sum_{j=0}^{k-1}\left(-1{)}^{j}(\begin{array}{c} -s\\ j \end{array}\right)\nabla^{j}\phi_{i+3},\;s=\frac{t-t_{i+3}}{h}, \end{array}$$

and substituting *dt* = *hds*, the corrector in the following form

$$\begin{array}{c} f_{c}^{\left(n-1\right)}\left(t_{i+3}\right)=f_{c}^{\left(n-1\right)}\left(t_{i}\right)+h\int_{-3}^{0}\sum_{j=0}^{k-1}\left(-1{)}^{j}(\begin{array}{c} -s\\ j \end{array}\right)\nabla^{j}\phi_{i+3}\;ds. \end{array}$$ (7)

Let denote

$$\begin{array}{c} \beta_{3,1,j}^{c}=(-1{)}^{j}\int_{-3}^{0}(\begin{array}{c} -s\\ j \end{array})ds, \end{array}$$ (8)

then by amending (7) we have,

$$\begin{array}{c} f_{c}^{\left(n-1\right)}\left(t_{i+3}\right)=f_{c}^{\left(n-1\right)}\left(t_{i}\right)+h\sum_{j=0}^{k-1}\beta_{3,1,j}^{c}\nabla^{j}\phi_{n+3}. \end{array}$$ (9)

Because derivation of subsequent orders follow the same sequence, we skip ahead to the *n*^*th*^ order derivation. The derivation continues with integrating (1) *n* number of times

$$\begin{array}{c} \begin{array}{cc} f_{c}\left(t_{i+3}\right)=f_{c}\left(t_{i}\right) & +hf_{c}^{\prime }\left(t_{i}\right)+\ldots +\frac{h^{\left(n-1\right)}}{(n-1)!}f_{c}^{\left(n-1\right)}\left(t_{i}\right)\\ & +h\int_{t_{i}}^{t_{i+3}}\frac{{\left(t_{i+3}-t\right)}^{\left(n-1\right)}}{(n-1)!}\phi (t,f,f^{\prime },\ldots ,f^{\left(n-1\right)})\;dt, \end{array} \end{array}$$

thus yielding

$$\begin{array}{c} f_{c}\left(t_{i+3}\right)=f_{c}\left(t_{i}\right)+hf_{c}^{\prime }\left(t_{i}\right)+\ldots +\frac{h^{\left(n-1\right)}}{(n-1)!}f_{c}^{\left(n-1\right)}\left(t_{i}\right)+h^{\left(n-1\right)}\sum_{j=0}^{k-1}\beta_{3,n,j}^{c}t^{j}\nabla^{j}\phi_{i}. \end{array}$$ (10)

after applying similar process as the preceding orders.

## Integration coefficients

In a three point block predictor-corrector method, it is highly beneficial to obtain the relationship between integration coefficients. In this section, firstly the explicit and implicit integration coefficients are derived. Then a recursive relationship between explicit and implicit integration coefficients and also coefficient of different orders are established.

### Explicit coefficients

To derive the explicit integration coefficient, we first denote the first order generating function, $G_{3,1}^{p}\left(t\right)$ as

$$\begin{array}{c} G_{3,1}^{p}\left(t\right)=\sum_{j=0}^{\infty }\beta_{3,1,j}^{p}t^{j}. \end{array}$$ (11)

By replacing $\beta_{3,1,j}^{p}t^{j}$ as defined in (4), $G_{3,1}^{p}\left(t\right)$ takes the following form

$$\begin{array}{c} G_{3,1}^{p}\left(t\right)=\sum_{j=0}^{\infty }(-t{)}^{j}\int_{0}^{3}(\begin{array}{c} -s\\ j \end{array})ds \end{array}$$ (12)

and by solving the integral establishes the following generating function

$$\begin{array}{c} G_{3,1}^{p}\left(t\right)=-[\frac{{\left(1-t\right)}^{-3}}{\text{log}(1-t)}-\frac{1}{\text{log}(1-t)}]. \end{array}$$ (13)

By way of (12), the generating functions can be written in terms of $\beta_{3,1,j}^{p}$

$$\begin{array}{c} \sum_{j=0}^{\infty }\beta_{3,1,j}^{p}t^{=}-[\frac{{\left(1-t\right)}^{-3}}{\text{log}(1-t)}-\frac{1}{\text{log}(1-t)}]. \end{array}$$ (14)

Expanding the functions in (14) then gives the following set of first order explicit coefficients,

$$\begin{array}{c} \beta_{3,1,0}^{p}=3,\;\beta_{3,1,k}^{p}=\frac{k(k+1)}{2}-\sum_{j=0}^{k-1}(\frac{\beta_{3,1,j}^{p}}{k-j+1}),\;k=1,2,\ldots . \end{array}$$

Next, the second order generating function has similar form as (11), where

$$\begin{array}{c} G_{3,2}^{p}\left(t\right)=\sum_{j=0}^{\infty }\beta_{3,2,j}^{p}t^{j}. \end{array}$$

and by substituting $\beta_{3,2,j}^{p}t^{j}$ with (5) we can rewrite $G_{3,2}^{p}\left(t\right)$ as

$$\begin{array}{c} G_{3,2}^{p}\left(t\right)=\sum_{j=0}^{\infty }(-t{)}^{j}\int_{0}^{3}\left(3-s\right)(\begin{array}{c} -s\\ j \end{array})ds. \end{array}$$ (15)

Then, solving (15) yields

$$\begin{array}{c} G_{3,2}^{p}\left(t\right)=[\frac{3}{\text{log}(1-t)}-\frac{G_{3,1}^{p}\left(t\right)}{\text{log}(1-t)}] \end{array}$$ (16)

thus establishing

$$\begin{array}{c} \beta_{3,2,0}^{p}=\beta_{3,1,1}^{p},\;\beta_{3,2,k}^{p}=\beta_{3,1,k+1}^{p}-\sum_{i=0}^{k-1}(\frac{\beta_{3,2,i}^{p}}{k-i+1}),\;k=1,2,\ldots . \end{array}$$

By mathematical induction, the *n*^*th*^ order explicit generating function and set of coefficients can be constructed as

$$\begin{array}{c} G_{3,n}^{p}\left(t\right)=\frac{1}{(n-1)!}[\frac{3^{\left(n-1\right)}}{\text{log}(1-t)}-\frac{\left(n-1\right)!G_{3,n-1}^{p}\left(t\right)}{\text{log}(1-t)}] \end{array}$$

and

$$\begin{array}{c} \beta_{3,n,0}^{p}=\beta_{3,n-1,1}^{p},\;\beta_{3,n,k}^{p}=\beta_{3,n-1,k+1}^{p}-\sum_{i=0}^{k-1}(\frac{\beta_{3,n,i}^{p}}{k-i+1}),\;k=1,2,\ldots . \end{array}$$

### Implicit coefficients

The implicit integration is derived by first defining the first order implicit generating function

$$\begin{array}{c} G_{3,1}^{c}\left(t\right)=\sum_{j=0}^{\infty }\beta_{3,1,j}^{c}t^{j}. \end{array}$$ (17)

Subsequent to defining the generating function, $G_{3,1}^{c}\left(t\right)$ is then equated as

$$\begin{array}{c} G_{3,1}^{c}\left(t\right)=\sum_{j=0}^{\infty }(-t{)}^{j}\int_{-3}^{0}(\begin{array}{c} -s\\ j \end{array})ds \end{array}$$

with the solution

$$\begin{array}{c} G_{3,1}^{c}\left(t\right)=-[\frac{1}{\text{log}(1-t)}-\frac{{\left(1-t\right)}^{3}}{\text{log}(1-t)}], \end{array}$$ (18)

hence extracting the following set of coefficients

$$\begin{array}{c} \beta_{3,1,0}^{c}=3,\;\beta_{3,1,1}^{c}=-3-\frac{\beta_{3,1,0}^{c}}{2},\;\beta_{3,1,2}^{c}=1-\sum_{j=0}^{1}(\frac{\beta_{3,1,j}^{c}}{3-j}), \end{array}$$

$$\begin{array}{c} \beta_{3,1,k}^{c}=-\sum_{j=0}^{k-1}(\frac{\beta_{3,1,j}^{c}}{k-j+1}),\;k=3,4\ldots . \end{array}$$

The second order implicit generating function is defined by

$$\begin{array}{c} G_{3,2}^{c}\left(t\right)=\sum_{j=0}^{\infty }\beta_{3,2,j}^{c}t^{j} \end{array}$$

where $\beta_{3,2,j}^{c}$ is replaced with

$$\begin{array}{c} \beta_{3,2,j}^{c}=(-1{)}^{j}\int_{-3}^{0}\left(-s\right)(\begin{array}{c} -s\\ j \end{array})ds \end{array}$$

thus re-defining the generating function as

$$\begin{array}{c} G_{3,2}^{c}\left(t\right)=\sum_{j=0}^{\infty }(-t{)}^{j}\int_{-3}^{0}\left(-s\right)(\begin{array}{c} -s\\ j \end{array})ds. \end{array}$$

Next, $G_{3,2}^{c}\left(t\right)$ is expressed **in** the form of

$$\begin{array}{c} G_{3,2}^{c}\left(t\right)=[\frac{3{\left(1-t\right)}^{3}}{\text{log}(1-t)}-\frac{G_{3,1}^{c}\left(t\right)}{\text{log}(1-t)}], \end{array}$$ (19)

which is then translated into the following set of coefficients

$$\begin{array}{c} \beta_{3,2,0}^{c}=\beta_{3,1,1}^{c},\;\beta_{3,2,k}^{c}=\beta_{3,1,k+1}^{c}-\sum_{j=0}^{k-1}(\frac{\beta_{3,1,j}^{c}}{k-j+1}),\;k=1,2,\ldots . \end{array}$$

The *n*^*th*^ order generating function can then be mathematically deduced as

$$\begin{array}{c} G_{3,n}^{c}\left(t\right)=\frac{1}{(n-1)!}[\frac{3{\left(1-t\right)}^{3}}{\text{log}(1-t)}-\frac{\left(n-1\right)!G_{3,1}^{c}\left(t\right)}{\text{log}(1-t)}], \end{array}$$

which subsequently produces the *n*^*th*^ order implicit coefficients

$$\begin{array}{c} \beta_{3,n,0}^{c}=\beta_{3,n-1,1}^{c},\;\beta_{3,n,k}^{c}=\beta_{3,n-1,k+1}^{c}-\sum_{j=0}^{k-1}(\frac{\beta_{3,n-1,j}^{c}}{k-j+1}),\;k=1,2,\ldots . \end{array}$$

### Recursive relationship

Calculating both predictor and corrector can be expensive, especially when the calculation requires large number of integration. By obtaining a recursive relationship between explicit and implicit coefficients, the corrector can be written in terms of predictor which will reduce the need for extensive calculation to obtain the corrected value. We begin by establishing the recursive interrelationship between explicit and implicit coefficients of the first order. From (13) and (18), the first order explicit and implicit generating functions are given respectively as

$$\begin{array}{c} G_{3,1}^{p}\left(t\right)=-[\frac{{\left(1-t\right)}^{-3}}{\text{log}(1-t)}-\frac{1}{\text{log}(1-t)}], \end{array}$$

and

$$\begin{array}{c} G_{3,1}^{c}\left(t\right)=-[\frac{1}{\text{log}(1-t)}-\frac{{\left(1-t\right)}^{3}}{\text{log}(1-t)}]. \end{array}$$

Next, consider rearranging the implicit first order generating function $G_{3,1}^{c}\left(t\right)$ which then can be denoted as

$$\begin{array}{c} G_{3,1}^{c}\left(t\right)=-{\left(1-t\right)}^{3}[\frac{{\left(1-t\right)}^{-3}}{\text{log}(1-t)}-\frac{1}{\text{log}(1-t)}] \end{array}$$ (20)

and then simplified as

$$\begin{array}{c} G_{3,1}^{c}\left(t\right)={\left(1-t\right)}^{3}G_{3,1}^{p}\left(t\right). \end{array}$$ (21)

Then substituting the set of first order explicit and implicit coefficients obtained in (11) and (17) into (21) establishes the following

$$\begin{array}{c} \sum_{j=0}^{\infty }\beta_{3,1,j}^{c}t^{j}={\left(1-t\right)}^{3}\sum_{j=0}^{\infty }\beta_{3,1,j}^{p}t^{j} \end{array}$$

which can reformulated yielding the following explicit-implicit relationship.

$$\begin{array}{c} \sum_{j=0}^{k}\frac{\left(k-j+1)(k-j+2\right)}{2}\beta_{3,1,j}^{c}=\beta_{3,1,k}^{p} \end{array}$$ (22)

We then continue with the second order explicit and implicit coefficients beginning with the generating functions which can be obtained respectively from (16) and (19) as

$$\begin{array}{c} G_{3,2}^{p}\left(t\right)=[\frac{3}{\text{log}(1-t)}-\frac{G_{3,1}^{p}\left(t\right)}{\text{log}(1-t)}] \end{array}$$

and

$$\begin{array}{c} G_{3,2}^{c}\left(t\right)=[\frac{3{\left(1-t\right)}^{3}}{\text{log}(1-t)}-\frac{G_{3,1}^{c}\left(t\right)}{\text{log}(1-t)}]. \end{array}$$

Using the relationship obtained from (21), $G_{3,2}^{c}\left(t\right)$ can be written as

$$\begin{array}{c} G_{3,2}^{c}\left(t\right)=[\frac{3{\left(1-t\right)}^{3}}{\text{log}(1-t)}-\frac{{\left(1-t\right)}^{3}G_{3,1}^{p}\left(t\right)}{\text{log}(1-t)}] \end{array}$$

and by way of (16), then denoted as

$$\begin{array}{c} G_{3,2}^{c}\left(t\right)={\left(1-t\right)}^{3}G_{3,2}^{p}\left(t\right) \end{array}$$

which yields a similar relationship as (22)

$$\begin{array}{c} \sum_{j=0}^{k}\frac{\left(k-j+1)(k-j+2\right)}{2}\beta_{3,2,j}^{c}=\beta_{3,2,k}^{p}. \end{array}$$

Finally via similar approach, the *n*^*th*^ order generating function can be mathematically deduced as

$$\begin{array}{c} G_{3,n}^{c}\left(t\right)=[\frac{3{\left(1-t\right)}^{3}}{\text{log}(1-t)}-\frac{{\left(1-t\right)}^{3}G_{3,n}^{p}\left(t\right)}{\text{log}(1-t)}] \end{array}$$

with the recursive relationship for *n*^*th*^ order explicit-implicit integration coefficient denoted as

$$\begin{array}{c} \sum_{j=0}^{k}\frac{\left(k-j+1)(k-j+2\right)}{2}\beta_{3,n,j}^{c}=\beta_{3,n,k}^{p}. \end{array}$$

## Order, stability and convergence

Conditions that satisfy order and zero stability of block methods have been researched by authors such as [29–31]. Order and stability of the proposed method presented in this study uses techniques provided in [30]. Before establishing order and stability of the method, here are preliminary definitions that are required.

### Preliminaries

This section provides necessary definitions to determine issue of stability, convergence and order of the method. We begin with the definition of the general linear multistep method.

**Definition 1** *The general linear multistep method is denoted by*

$$\begin{array}{c} \sum_{j=0}^{k}\alpha_{j}y_{n+j}=h\sum_{j=0}^{k}\beta_{j}g_{n+j} \end{array}$$

Next, lets define the linear differential operator for the general linear multistep method as

**Definition 2** *The linear differential operator L associated with the linear multistep method is defined*

$$L[g(t);h]≔\sum_{i=0}^{k}\left[\alpha_{i}g(t+ih)\right]-h\beta_{i}g\prime (t+ih)],$$

*where g*(*t*) *is an arbitrary function in C*^1^[*a*, *b*].

Let *g*(*t*) be a function that is *q* times differentiable. Next, expand *g*(*t* + *ih*) and *g*′(*t* + *ih*) about *t* and arrange as

$$\begin{array}{c} L\left[g\left(t\right);h\right]:=C_{0}g\left(t\right)+C_{1}hg^{\prime }\left(t\right)+\ldots +C_{q}h^{q}g^{\left(q\right)}\left(t\right) \end{array}$$

where *C*_*i*_, *i* = 0, 1.…, *q*, … are constants.

**Definition 3** *The linear multistep method and associated difference operator L are said to be of order p if, C*_0_ = *C*_1_ = … = *C*_*q*_ = 0, *C*_*q*+1_ ≠ 0 where

$$\begin{array}{c} \begin{array}{cc} C_{0} & =\alpha_{0}+\alpha_{1}+\ldots +\alpha_{k}\\ C_{1} & =\left(\alpha_{1}+2\alpha_{2}+\ldots +k\alpha_{k}\right)-\left(\beta_{1}+\beta_{2}+\ldots +\beta_{k}\right)\\ \vdots & \;\;\vdots \\ C_{p} & =\frac{1}{p}\left(\alpha_{1}+2\alpha_{2}^{p}+\ldots +k^{p}\alpha_{k}\right)-\frac{1}{q-1}\left(\beta_{1}+2^{p-1}\beta_{2}+\ldots +k^{p-1}\beta_{k}\right) \end{array} \end{array}$$

**Definition 4** *The block method is zero stable if the roots r*_*j*_, *j* = 1, …*k of the first characteristic polynomial ρ*(*r*) *denoted by*

$$\begin{array}{c} \rho \left(r\right)=\text{det}(\sum_{i=0}^{m}A_{i}r^{m-i})=0 \end{array}$$

*satisfies the conditions* |*r*_*j*_| ≤ 1 *and the roots with* |*r*_*j*_| = 1 *where the multiplicity does not exceed 2*.

**Definition 5** *A Linear Multistep Method is said to be consistent if the LLM is of order p* ≥ 1.

### Order of the method

In the current section, order of both predictor and corrector will be substantiated for *k* = 6 number of back values. For simplicity, the example selected is of *f*′. Beginning with the predictor, *f*_*i*+*b*_ for *b* = 1, 2, 3 of the three block method can be expressed as

$$\begin{array}{c} f_{i+1}=f_{i}+h(\frac{1901}{720}\phi_{i}-\frac{1387}{360}\phi_{i-1}+\frac{109}{30}\phi_{i-2}-\frac{637}{360}\phi_{i-3}+\frac{251}{720}\phi_{i-4}) \end{array}$$ (23)

$$\begin{array}{c} f_{i+2}=f_{i}+h(\frac{1079}{90}\phi_{i}-\frac{1198}{45}\phi_{i-1}+\frac{424}{15}\phi_{i-2}-\frac{658}{45}\phi_{i-3}+\frac{269}{90}\phi_{i-4}) \end{array}$$ (24)

$$\begin{array}{c} f_{i+3}=f_{i}+h(\frac{2877}{80}\phi_{i}-\frac{3819}{40}\phi_{i-1}+\frac{1089}{10}\phi_{i-2}-\frac{2349}{40}\phi_{i-3}+\frac{987}{80}\phi_{i-4}) \end{array}$$ (25)

The order of the method follows Definition 1, where (23) to (25) are defined as matrices which satisfy

$$\begin{array}{c} \sum_{j=0}^{3}A_{j}F_{m+j-3}^{p}=h\sum_{j=0}^{3}B_{j}\Phi_{m+j-3}^{p}, \end{array}$$

where the matrices obtained are as follows

$$\begin{array}{c} \begin{array}{cc} \sum_{j=0}^{3}A_{j}F_{m+j-3}^{p}= & \left(\begin{array}{ccc} 0 & 0 & 0\\ 0 & 0 & 0\\ 0 & 0 & 0 \end{array})(\begin{array}{c} f_{i-5}\\ f_{i-4}\\ f_{i-3} \end{array})+(\begin{array}{ccc} 0 & 0 & -1\\ 0 & 0 & -1\\ 0 & 0 & -1 \end{array})(\begin{array}{c} f_{i-2}\\ f_{i-1}\\ f_{i} \end{array}\right)\\ & +(\begin{array}{ccc} 1 & 0 & 0\\ 0 & 1 & 0\\ 0 & 0 & 1 \end{array})(\begin{array}{c} f_{i+1}\\ f_{i+2}\\ f_{i+3} \end{array}) \end{array} \end{array}$$

and

$$\begin{array}{c} \begin{array}{cc} \sum_{j=0}^{3}B_{j}\Phi_{m+j-3}^{p}= & (\begin{array}{ccc} 0 & \frac{251}{720} & -\frac{637}{360}\\ 0 & \frac{269}{90} & -\frac{658}{45}\\ 0 & \frac{987}{80} & -\frac{2349}{40} \end{array})(\begin{array}{c} \phi_{i-5}\\ \phi_{i-4}\\ \phi_{i-3} \end{array})+(\begin{array}{ccc} \frac{109}{30} & -\frac{1387}{360} & \frac{1901}{720}\\ \frac{424}{15} & -\frac{1198}{45} & \frac{1079}{90}\\ \frac{1089}{10} & -\frac{3819}{40} & \frac{2877}{80} \end{array})\times \\ & \left(\begin{array}{c} \phi_{i-2}\\ \phi_{i-1}\\ \phi_{i} \end{array})+(\begin{array}{ccc} 0 & 0 & 0\\ 0 & 0 & 0\\ 0 & 0 & 0 \end{array})(\begin{array}{c} \phi_{i+1}\\ \phi_{i+2}\\ \phi_{i+3} \end{array}\right) \end{array} \end{array}$$

The coefficients matrices are then split into sets of vector columns

$$\begin{array}{l} a_{0}=\left(\begin{array}{c} 0\\ 0\\ 0 \end{array}\right),\;a_{1}=\left(\begin{array}{c} 0\\ 0\\ 0 \end{array}\right),\;a_{2}=\left(\begin{array}{c} 0\\ 0\\ 0 \end{array}\right),\;a_{3}=\left(\begin{array}{c} 0\\ 0\\ 0 \end{array}\right),\;a_{4}=\left(\begin{array}{c} 0\\ 0\\ 0 \end{array}\right),\\ a_{5}=\left(\begin{array}{c} -1\\ -1\\ -1 \end{array}\right),\;a_{6}=\left(\begin{array}{c} 1\\ 0\\ 0 \end{array}\right),\;a_{7}=\left(\begin{array}{c} 0\\ 1\\ 0 \end{array}\right),\;a_{8}=\left(\begin{array}{c} 0\\ 0\\ 1 \end{array}\right) \end{array}$$

$$\begin{array}{l} b_{0}=\left(\begin{array}{c} 0\\ 0\\ 0 \end{array}\right),\;b_{1}=\left(\begin{array}{c} \frac{251}{720}\\ \frac{269}{90}\\ \frac{987}{80} \end{array}\right),\;b_{2}=\left(\begin{array}{c} -\frac{637}{360}\\ -\frac{658}{45}\\ -\frac{2349}{40} \end{array}\right),\;b_{3}=\left(\begin{array}{c} \frac{109}{30}\\ \frac{424}{15}\\ \frac{1089}{10} \end{array}\right),\\ b_{4}=\left(\begin{array}{c} -\frac{1387}{360}\\ -\frac{1198}{45}\\ -\frac{3819}{40} \end{array}\right),\;b_{5}=\left(\begin{array}{c} \frac{1901}{720}\\ \frac{1079}{90}\\ \frac{2877}{80} \end{array}\right),\;b_{6}=\left(\begin{array}{c} 0\\ 0\\ 0 \end{array}\right),\;b_{7}=\left(\begin{array}{c} 0\\ 0\\ 0 \end{array}\right),\;b_{8}=\left(\begin{array}{c} 0\\ 0\\ 0 \end{array}\right) \end{array}$$

By way of Definition 3, from the set of vector columns yield

$$C_{0}=C_{1}=C_{2}=\ldots =C_{5}=\left(\begin{array}{c} 0\\ 0\\ 0 \end{array}\right),\;C_{6}=\left(\begin{array}{c} \frac{95}{288}\\ \frac{33}{10}\\ \frac{2499}{160} \end{array}\right)$$

thus, establishing the predictor is of order 5 with a corresponding error constant of

$$C_{6}=\left(\begin{array}{c} \frac{95}{288}\\ \frac{33}{10}\\ \frac{2499}{160} \end{array}\right)$$

Next, the order method for the corrector begins by denoting the corrector as follows

$$\begin{array}{c} f_{i+1}=f_{i}+h(\frac{95}{288}\phi_{i+1}+\frac{1427}{1440}\phi_{i}-\frac{133}{240}\phi_{i-1}+\frac{241}{720}\phi_{i-2}-\frac{173}{1440}\phi_{i-3}+\frac{3}{160}\phi_{i-4}) \end{array}$$ (26)

$$\begin{array}{c} f_{i+2}=f_{i}+h(\frac{14}{45}\phi_{i+2}+\frac{43}{30}\phi_{i+1}+\frac{7}{45}\phi_{i}+\frac{14}{90}\phi_{i-1}-\frac{6}{90}\phi_{i-2}+\frac{1}{90}\phi_{i-3}) \end{array}$$ (27)

$$\begin{array}{c} f_{i+3}=f_{i}+h(\frac{51}{160}\phi_{i+3}+\frac{219}{160}\phi_{i+2}+\frac{57}{80}\phi_{i+1}+\frac{57}{80}\phi_{i}-\frac{21}{160}\phi_{i-1}+\frac{3}{160}\phi_{i-2}) \end{array}$$ (28)

Again by Definition 3 and derived in similar fashion as the predictor, it is established that the corrector formula is of order 6 with the error constant

$$C_{7}=\left(\begin{array}{c} -\frac{863}{60480}\\ -\frac{37}{3780}\\ -\frac{90}{2240} \end{array}\right)$$

### Zero stability

Zero stability governs certain conditions which dictates the viability of a linear multistep method. The stability of the three point block method can be established by following similar conditions as presented in [7]. To establish whether the method is zero stable, derivation begins with the predictor. By way of the standard linear test problem

$$\begin{array}{c} f^{\prime }=\lambda f. \end{array}$$

and applying Eqs (23) to (25) and (26) to (28), the predictor and corrector can reformulated as

$$\begin{array}{c} \begin{array}{cc} \left(\begin{array}{ccc} 1 & 0 & 0\\ 0 & 1 & 0\\ 0 & 0 & 1 \end{array})(\begin{array}{c} f_{i+1}\\ f_{i+2}\\ f_{i+3} \end{array}\right) & =(\begin{array}{ccc} 0 & 0 & 1\\ 0 & 0 & 1\\ 0 & 0 & 1 \end{array})(\begin{array}{c} f_{i-2}\\ f_{i-1}\\ f_{i} \end{array})+(\begin{array}{ccc} \frac{109}{30} & -\frac{1387}{360} & \frac{1901}{720}\\ \frac{424}{15} & -\frac{1198}{45} & \frac{1079}{90}\\ \frac{1089}{10} & -\frac{3819}{40} & \frac{2877}{80} \end{array})\times \\ & \times (\begin{array}{c} \phi_{i-2}\\ \phi_{i-1}\\ \phi_{i} \end{array})h\lambda +(\begin{array}{ccc} 0 & \frac{251}{720} & -\frac{637}{360}\\ 0 & \frac{269}{90} & -\frac{658}{45}\\ 0 & \frac{987}{80} & -\frac{2349}{40} \end{array})(\begin{array}{c} \phi_{i-5}\\ \phi_{i-4}\\ \phi_{i-3} \end{array})h\lambda \end{array} \end{array}$$ (29)

and

$$\begin{array}{c} \begin{array}{cc} \left(\begin{array}{ccc} 1 & 0 & 0\\ 0 & 1 & 0\\ 0 & 0 & 1 \end{array})(\begin{array}{c} f_{i+1}\\ f_{i+2}\\ f_{i+3} \end{array}\right) & =(\begin{array}{ccc} 0 & 0 & 1\\ 0 & 0 & 1\\ 0 & 0 & 1 \end{array})(\begin{array}{c} f_{i-2}\\ f_{i-1}\\ f_{i} \end{array})+(\begin{array}{ccc} \frac{95}{288} & 0 & 0\\ \frac{43}{30} & \frac{14}{45} & 0\\ \frac{57}{80} & \frac{219}{160} & \frac{51}{160} \end{array})\times \\ & \times (\begin{array}{c} \phi_{i+1}\\ \phi_{i+2}\\ \phi_{i+3} \end{array})h\lambda +(\begin{array}{ccc} \frac{241}{720} & -\frac{133}{240} & \frac{1427}{1440}\\ -\frac{6}{90} & \frac{14}{90} & \frac{7}{45}\\ \frac{3}{160} & -\frac{21}{160} & \frac{57}{80} \end{array})(\begin{array}{c} \phi_{i-2}\\ \phi_{i-1}\\ \phi_{i} \end{array})h\lambda \\ & +(\begin{array}{ccc} 0 & \frac{3}{160} & -\frac{173}{1440}\\ 0 & 0 & \frac{1}{90}\\ 0 & 0 & 0 \end{array})(\begin{array}{c} \phi_{i-5}\\ \phi_{i-4}\\ \phi_{i-3} \end{array})h\lambda \end{array} \end{array}$$ (30)

respectively. Next, consider the stability polynomial which is denoted by,

$$\begin{array}{c} \rho \left(t;\Lambda \right)=\text{det}(\sum_{i=0}^{m}A_{i}t^{m-i}),\;\Lambda =h\lambda . \end{array}$$

By way of the stability polynomial, (29) and (30) can consequently be expressed as

$$\begin{array}{c} \begin{array}{cc} \rho_{p}\left(t;\Lambda \right)= & t^{6}-(1+\frac{9341}{720}\Lambda )t^{5}+(\frac{15319}{360}\Lambda^{2}+\frac{1739}{90}\Lambda )t^{4}\\ & -(\frac{7661}{80}\Lambda^{3}+\frac{11759}{360}\Lambda^{2}+\frac{6731}{720}\Lambda )t^{3}-(\frac{197}{24}\Lambda^{3}+\frac{901}{72}\Lambda^{2})t^{2}\\ & -\frac{37}{240}t\Lambda^{3}t \end{array} \end{array}$$

and

$$\begin{array}{c} \begin{array}{cc} \rho_{c}\left(t;\Lambda \right)= & (1-\frac{2261}{69120}\Lambda^{3}+\frac{636457}{2073600}\Lambda^{2}-\frac{691}{720}\Lambda )t^{6}\\ & -(1+\frac{14032177}{6912000}\Lambda^{3}+\frac{516739}{691200}\Lambda^{2}+\frac{1903}{720}\Lambda )t^{5}\\ & -(\frac{2359}{54000}\Lambda^{3}+\frac{14939}{414720}\Lambda^{2})t^{4}-(\frac{7661}{80}\Lambda^{3}+\frac{11759}{360}\Lambda^{2}+\frac{6731}{720}\Lambda )t^{3}\\ & -(\frac{49}{64000}\Lambda^{3}+\frac{41}{25600}\Lambda^{2})t^{2}-\frac{1}{256000}t\Lambda^{3}t \end{array} \end{array}$$

For zero stability, we let Λ = 0 which yields the stability polynomial for both predictor and corrector as

$$\begin{array}{c} \rho_{p}\left(t;0\right)=\rho_{c}\left(t;0\right)=t^{6}-t^{5} \end{array}$$

with roots *t*_0_ = … = *t*_5_ = 0, *t*_6_ = 1. Thus, Definition 4 dictates that both predictor and corrector formulae are zero stable.

## Convergence of the backward difference method

Works of [32] highlights that certain conditions are required in order to validate the convergence of a backward difference formulation. Those conditions are governed by the following.

**Theorem 1** *Conditions necessary for the linear multistep method* (6) *and* (10) *to be convergent are*

- Methods must be consistent
- Methods must be zero stable

For proof of the theorem, we refer readers to works of [32].

The order of the method was established in earlier subsection as following: The predictor

$$\begin{array}{c} C_{0}=\ldots =C_{5}=(\begin{array}{c} 0\\ 0\\ 0 \end{array}),\;C_{6}\neq (\begin{array}{c} 0\\ 0\\ 0 \end{array}): \end{array}$$

The corrector

$$\begin{array}{c} C_{0}=\ldots =C_{6}=(\begin{array}{c} 0\\ 0\\ 0 \end{array}),\;C_{7}\neq (\begin{array}{c} 0\\ 0\\ 0 \end{array}): \end{array}$$

Hence, by Definition 5 the predictor and corrector are consistent of orders 5 and 6 respectively. And as shown in the previous subsection, both methods are zero stable ergo satisfying the necessary conditions for convergence.

Finally, we proceed with the subsequent section, the long awaited numerical results.

## Results and discussion

Real life science and engineering problems are often modeled in the form of ODEs. Every so often, these problems are not able to be solved analytically, thus requires numerical approximations. These numerical approximations are often used as alternative solutions due to the absence of analytical solution. The current section provides results for selected higher order ODEs using the proposed three point block method (3PBCS) with constant step size. Problems selected varies from linear to nonlinear artificial and real life problems which are in the form of single and systems of equations. The problems selected also consist of various difficulty levels in order to provide a holistic overview of the 3PBCS method’s capability. For a more just comparison, the 3PBCS method is compared against Direct Integration (DI), one point block (1PBCS) and two point block (2PBCS) methods. Results will be compared in terms of accuracy, function evaluations and efficiency. The error solution used in this study adopts techniques suggested in [12] which is given by

$$\begin{array}{c} {\text{ErrMax}}_{n}=|\frac{{\left(f_{i}\right)}_{n}-f{\left(t_{i}\right)}_{n}}{A+B(f{\left(t_{i}\right)}_{n})}|. \end{array}$$ (31)

where the *n*^*th*^ component of the exact solution is denoted by (*f*_*i*_)_*n*_ and the *n*^*th*^ component of the approximated solution for *f* is denoted by *f*(*t*_*i*_)_*n*_. The error Err_*n*_ is used to define three types of error test used this study, absolute error (*A* = 1, *B* = 0), relative error (*A* = 0, *B* = 1) and mixed error (*A* = 1, *B* = 1) tests. Before continuing with the numerical results, these are a few terms that will be used throughout the section.

### Higher order problems

This section begins with higher order differential problems which are artificial in nature and of different orders then followed with well known differential equations. Examples 1-3 are 4^*th*^ to 5^*th*^ order problems obtained from various sources.

*Example 1*: A non homogeneous 4^*th*^ order ODE,

$$\begin{array}{c} f^{\left(iv\right)}=f^{2}+{\text{cos}}^{2}\left(t\right)+\text{sin}\left(t\right)-1,\;0\leq t\leq 10 \end{array}$$

with the initial conditions *f*|_0_ = 0, *f*′|_0_ = 1, *f*″|_0_ = 0, *f*‴|_0_ = −1 and given analytical solution *f*(*t*) = sin(*t*). (Source: [33])

*Example 2*: A 4^*th*^ order non linear ODE

$$\begin{array}{c} f^{\left(iv\right)}=0.09(\frac{f^{\prime \prime 2}f^{\prime }f}{f^{\prime \prime \prime }}+f^{\prime \prime }f^{2}),\;0\leq t\leq 10 \end{array}$$

with the initial condtions *f*|_0_ = 1, *f*′|_0_ = −0.1, *f*″|_0_ = 0.02, *f*‴|_0_ = −0.006 and given analytical solution $f\left(t\right)=\frac{10}{10+t}$. (Source: [34])

*Example 3*: A 5^*th*^ order non linear ODE

$$\begin{array}{c} f^{\left(v\right)}=6(2f^{\prime 3}+6ff^{\prime }f^{\prime \prime }+f^{2}f^{\prime \prime \prime }),\;1\leq t\leq 3 \end{array}$$

with the initial condtions *f*|_1_ = 1, *f*′|_1_ = −1, *f*″|_1_ = 2, *f*‴|_1_ = −6*f*^(*iv*)^|_1_ = 24 and given analytical solution $f\left(t\right)=\frac{1}{t}$. (Source: [16])

**Fig 1:**
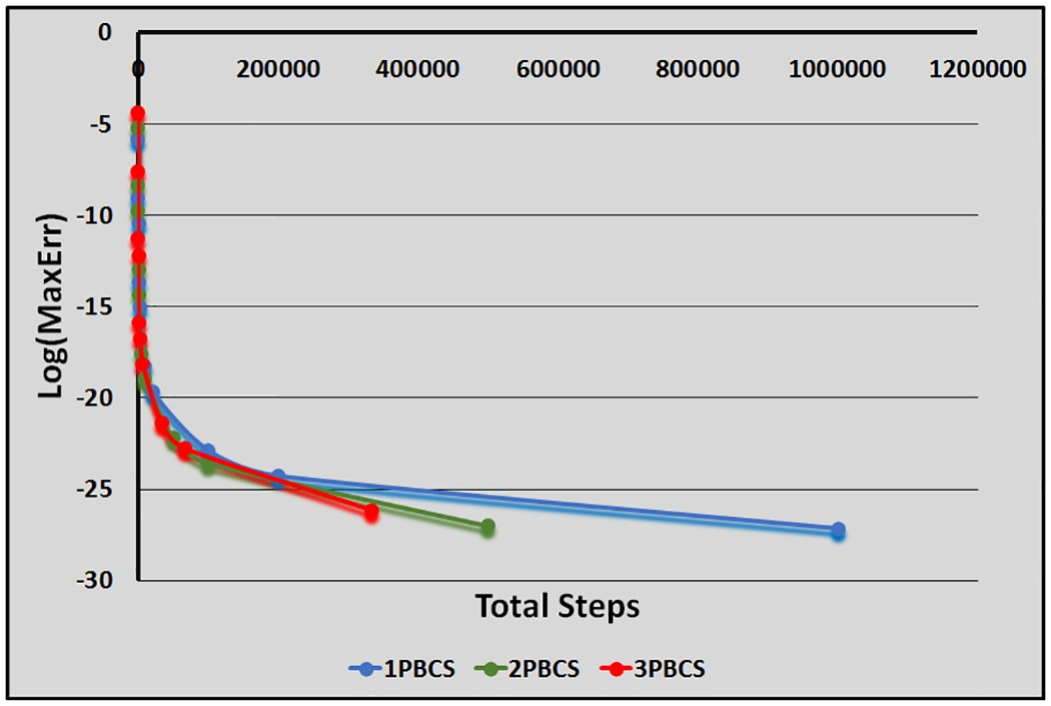
Efficiency of the 1PBCS, 2PBCS and 3PBCS methods for Example 1.

**Fig 2:**
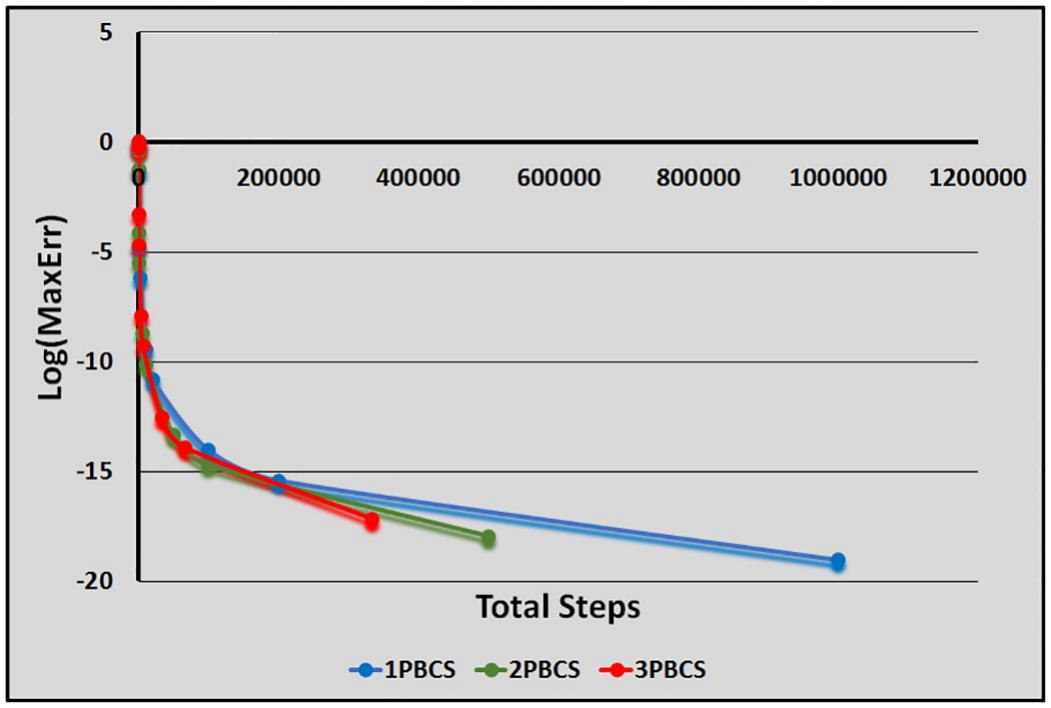
Efficiency of the 1PBCS, 2PBCS and 3PBCS methods for Example 2.

**Fig 3:**
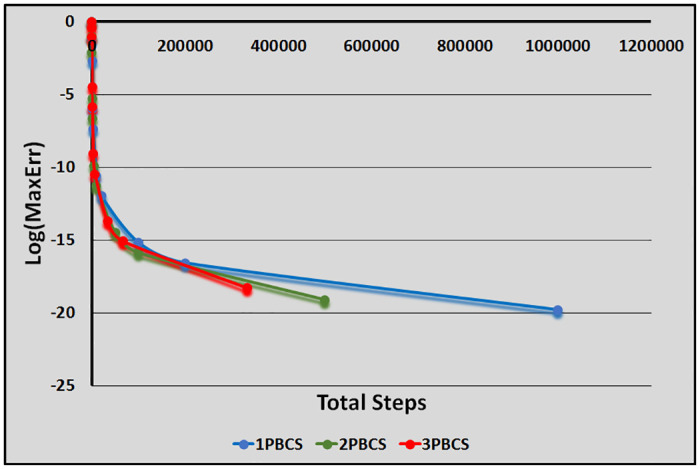
Efficiency of the 1PBCS, 2PBCS and 3PBCS methods for Example 3.

Table 1 presents numerical results of the 1PBCS, 2PBCS and 3PBCS methods for Examples 1 -3. The 1PBCS approximates single steps of equidistant, the 2PBCS approximates two-points of equidistant simultaneously and the 3PBCS approximates three-points of equidistant simultaneously. Results will compare accuracy and total steps approximated by all three methods for step sizes 10^−1^, 10^−2^, 10^−3^, 10^−4^ and 10^−5^. Tstep represents the total steps required and Err denotes the maximum error estimated for each method in the interval *T*_0_ ≤ *t* ≤ *T*_*n*_. The 1PBCS and 2PBCS methods were selected to compare efficiency of the 3PBCS when solving higher order ODEs with other backward difference methods to provide a fair analysis. The analysis of Examples 1 to 3 begins with results provided in Table 1. As shown in Table 1, the 3PBCS method obviously requires less calculation steps compared to the latter even though some loss of accuracy is expected. For Example 2, the 3PBCS is able to match the level of accuracy of 1PBCS and 2PBCS methods but slightly under-performed in Examples 1 and 3. Whereas Table 2 provides computational time (Time) which is recorded in seconds for Examples 1-3. As exhibited in Table 2, 3PBCS requires less computational time compared to 1PBCS and 2PBCS methods for almost all step sizes, except at *H* = 1 × 10^−1^. This is due to the initial calculation of the three-point block integration coefficients. The small amount of Tsteps required to calculate the solutions from beginning to end outweighs the efficiency of the 3PBCS method. But as a smaller *H* is selected, more Tstep is required which redistributes calculation time of the initial 3PBCS integration coefficients thus leads to a lesser overall computational time. This loss is barely significant and can be overcome in a variable order stepsize algorithm. Figs 1–3 compares the efficiency of the methods. As defined in [12], the efficiency of the methods is the accuracy per step and by way of the under most curve, graphs depicted in Figs 1–3 show the 3PBCS method to be slightly more efficient than latter methods.

**Table 1:** Comparison between 1PBCS, 2PBCS dan 3PBCS methods for Examples 1 to 3.

H	Method	Example 1		Example 2		Example 3	
		TSteps	ErrMax	TSteps	ErrMax	TSteps	ErrMax
10^−1^	1PBCS	100	7.45376e-01	100	1.15401e-04	20	3.21086e-01
	2PBCS	50	6.66935e-01	50	2.34228e-04	10	3.59297e-01
	**3PBCS**	**34**	**9.35380e-01**	**34**	**5.11552e-04**	**7**	**6.48511e-01**
10^−2^	1PBCS	1000	8.23960e-03	1000	1.15142e-06	200	2.54695e-03
	2PBCS	500	1.62497e-02	500	2.30738e-06	100	5.16502e-03
	**3PBCS**	**334**	**3.79323e-02**	**334**	**5.17431e-06**	**67**	**1.15395e-02**
10^−3^	1PBCS	10000	8.16835e-05	10000	1.15118e-08	2000	2.54180e-05
	2PBCS	5000	1.63524e-04	5000	2.30283e-08	1000	5.09487e-05
	**3PBCS**	**3334**	**3.67387e-04**	**3334**	**5.17962e-08**	**667**	**1.14395e-04**
10^−4^	1PBCSI	100000	8.16543e-07	100000	1.14942e-10	20000	2.54175e-07
	2PBCS	50000	1.63373e-06	50000	2.30188e-10	10000	5.08467e-07
	**3PBCS**	**33334**	**3.67505e-06**	**33334**	**5.18013e-10**	**6667**	**1.14379e-06**
10^−5^	1PBCS	1000000	5.45040e-09	1000000	1.58909e-12	200000	2.54041e-09
	2PBCS	500000	1.62002e-08	500000	1.86703e-12	100000	5.08386e-09
	**3PBCS**	**333334**	**3.58752e-08**	**333334**	**4.42069e-12**	**66667**	**1.14377e-08**

**Table 2:** Comparison in computational time between 1PBCS, 2PBCS dan 3PBCS methods for Examples 1 to 3.

H	Method	Example 1	Example 2	Example 3
		Time	Time	Time
10^−1^	1PBCS	3.032	3.032	3.027
	2PBCS	3.032	3.032	3.027
	**3PBCS**	**3.033**	**3.033**	**3.029**
10^−2^	1PBCS	3.044	3.041	3.036
	2PBCS	3.041	3.037	3.036
	**3PBCS**	**3.037**	**3.035**	**3.036**
10^−3^	1PBCS	3.049	3.048	3.052
	2PBCS	3.046	3.046	3.052
	**3PBCS**	**3.042**	**3.041**	**3.049**
10^−4^	1PBCSI	3.182	3.192	3.072
	2PBCS	3.174	3.186	3.070
	**3PBCS**	**3.165**	**3.175**	**3.068**
10^−5^	1PBCS	4.311	4.322	3.289
	2PBCS	4.301	4.310	3.285
	**3PBCS**	**4.289**	**4.298**	**3.278**

*Example 4*: A 3^*rd*^ order systems of ODE,

$$\begin{array}{c} f_{1}^{\prime \prime \prime }=\frac{1}{2}e^{4t}f_{3}f_{2}^{\prime },\;f_{2}^{\prime \prime \prime }=\frac{8}{3}e^{2t}f_{1}f_{3}^{\prime },\;f_{3}^{\prime \prime \prime }=27f_{2}f_{1}^{\prime },\;0\leq t\leq 3 \end{array}$$

with the initial conditions *f*_1_|_0_ = 1, $f_{1}^{\prime }{|}_{0}=-1$, $f_{1}^{\prime \prime }{|}_{0}=1$, *f*_2_|_0_ = 1, $f_{2}^{\prime }{|}_{0}=-2$, $f_{2}^{\prime \prime }{|}_{0}=4$, *f*_3_|_0_ = 1, $f_{3}^{\prime }{|}_{0}=-3$ and $f_{3}^{\prime \prime }{|}_{0}=9$ and given analytical solutions *f*_1_(*t*) = *e*^−*t*^, *f*_2_(*t*) = *e*^−2*t*^ and *f*_3_(*t*) = *e*^−3*t*^. (Source: [35])

Table 3 compares results between the DI and 3PBCS methods. Opposed to the 3PBCS backward difference formulation, the DI method is derived with a divided difference formulation. The DI method was chosen to test the 3PBCS with a competing predictor-corrector multistep method. Results presented highlights maximum error (ErrEQ1, ErrEQ2, ErrEQ3) for each of equation, *f*_1_, *f*_2_ and *f*_3_ and the overall maximum error for each step size, ErrMax. The error is obtained by comparing approximated solution with the exact solution, $\left|\frac{{\left(f_{i}\right)}_{n}-f{\left(t_{i}\right)}_{n}}{A+B(f{\left(t_{i}\right)}_{n})}\right|$ with conditions established in (31). The DI was selected because of its divided difference formulation. The purpose was to test the performance of the 3PBCS method against an alternative multistep method using similar parameter. In the current case, both methods were set using similar order methods (same number of back values). Results of the current example clearly illustrates the advantage of 3PBCS over the DI method.

**Table 3:** Comparison between DI dan 3PBCS method for Example 4.

H	Method	TSteps	ErrEQ1	ErrEQ2	ErrEQ3	ErrMax
10^−1^	DI	30	1.00000e+00	1.00000e+00	1.00000e+00	1.00000e+00
	**3PBCS**	**10**	**1.00000e+00**	**1.00000e+00**	**1.00000e+00**	**1.00000e+00**
10^−2^	DI	300	1.00000e+00	1.00000e+00	1.00000e+00	1.00000e+00
	**3PBCS**	**100**	**6.69108e-02**	**1.77349e-02**	**1.05606e-02**	**6.69108e−02**
10^−3^	DI	3000	8.40056e−01	3.82868e−01	1.15686e−01	8.40056e−01
	**3PBCS**	**1000**	**1.98994e-04**	**1.78543e-04**	**1.22428e-04**	**1.98994e-04**
10^−4^	DI	30000	4.04766e−02	1.56457e−02	2.34364e−03	4.04766e−02
	**3PBCS**	**10000**	**1.97238e-06**	**1.78345e-06**	**1.22641e-06**	**1.97238e-06**
10^−5^	DI	300000	2.31051e−03	1.55405e−03	2.24310e−04	2.31051e−03
	**3PBCS**	**100000**	**1.97209e-08**	**1.78295e-08**	**1.22651e-08**	**1.97209e-08**

*Example 5*: The problem presented is a artificial second order ODE with periodic solution. The second order ODE

$$\begin{array}{c} f^{\prime \prime }=-2500f+1100\;\text{cos}\left(60t\right) \end{array}$$

with the initial condtions *f*|_0_ = −1, *f*′|_0_ = 50 and given analytical solution *f*(*t*) = sin(50*t*) − cos(60*t*) provides an unique oscillatory solution.

**Fig 4:**
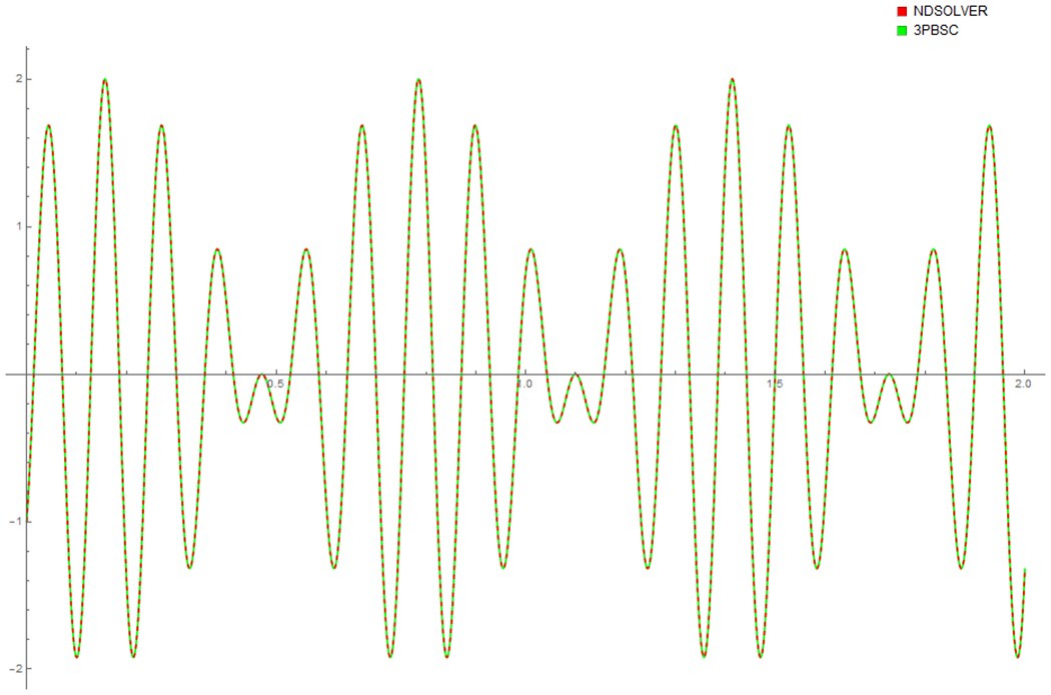
Comparison between NDSOLVER and 3PBCS for the solution of Example 5.

**Fig 5:**
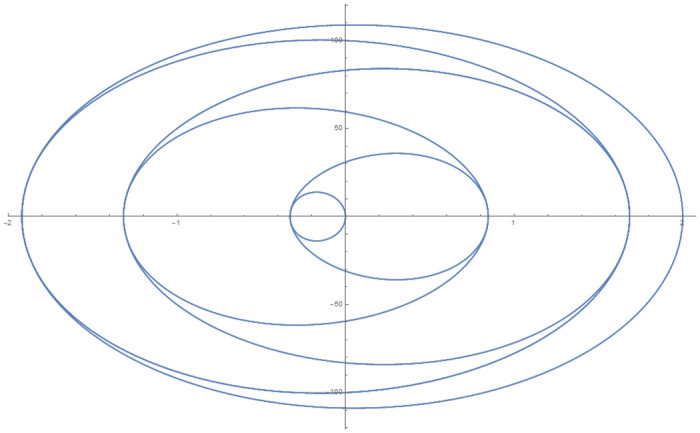
Parametric plot of *f* and *f*′ of 3PBCS method for Example 5.

**Fig 6:**
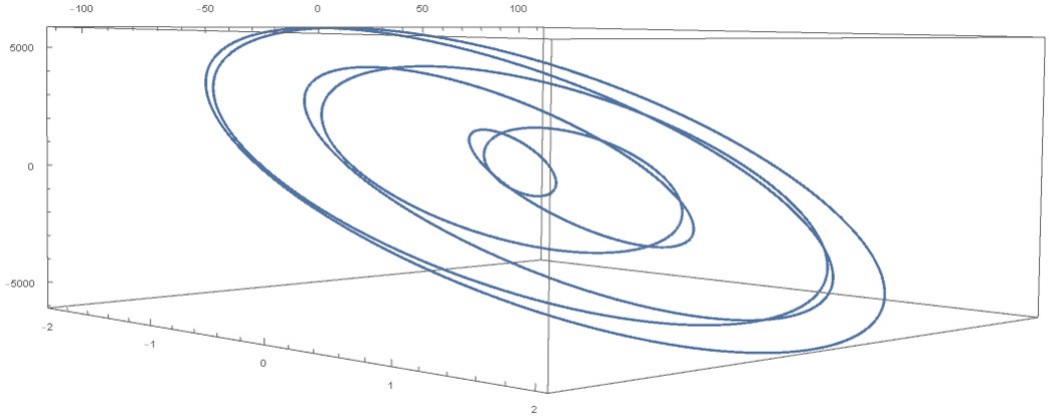
3D parametric plot of *f*, *f*′ and *f*″ approximated by 3PBCS method for Example 5.

**Fig 7:**
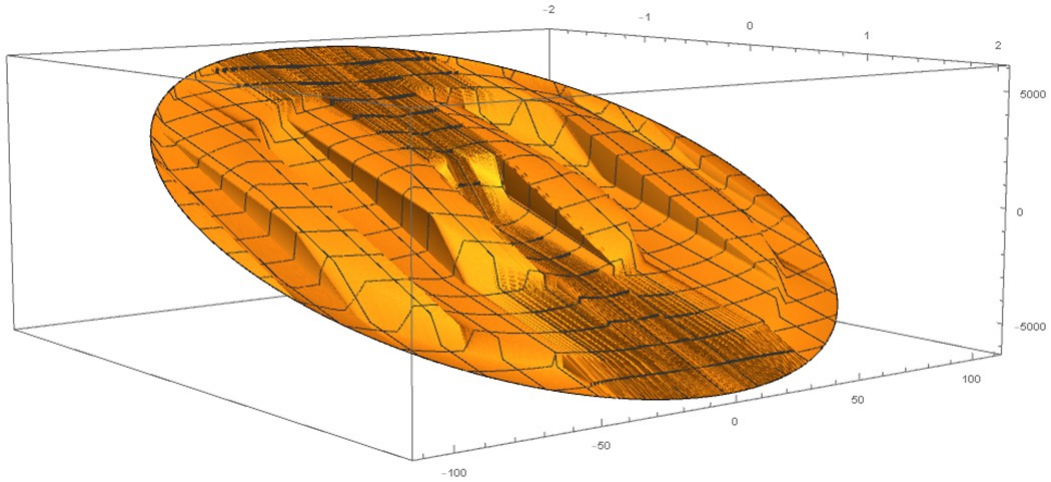
3D representation of 3PBCS method for Example 5.

Table 4 provides numerical approximation by the 3PBCS and the provided exact solution for Example 5. Due to the difficulty of the current example, the step size of order *H* = 10^−5^ is selected. Results show a consistent approximation error of 1×10^−6^ for every point. To further validate the accuracy of 3PBCS as suitable method for the approximation of a non linear ODE with periodic solution, 3PBCS is compared against NDSOLVER, a preset Euler method equipped in Mathematica 12. The plotted graph displayed in Fig 4 shows that the 3PBCS method matches the solution obtained by NDSOLVER thus, confirming the accuracy of the method. Figs 5 and 6 are 2D and 3D parametric plots of Example 5 by the 3PBCS. For a clear understanding of the difficulty level for Example 5, readers may also refer to a 3D representation of the periodic solutions as illustrated in Fig 7.

**Table 4:** Comparison between the 3PBCS approximation and exact solution for Example 5.

	Approximate *f*(*t*_*n*+3_)	Exact *f*_*n*_(*t*)	ErrMax
0.5	-2.86605e−03	-2.86603e−03	1.29539e−06
1	6.90035e−01	6.90038e−01	2.46112e−06
1.5	6.02881e−02	6.02920e−02	3.85117e−06
2	-1.32055e+00	-1.32055e+00	4.36827e−06

### Application problems

Test problems in the current section are practical ODEs that are found in real applications including two body motion, thin flow and electrical circuits. Lets begin with the renowned two body motion.

**Fig 8:**
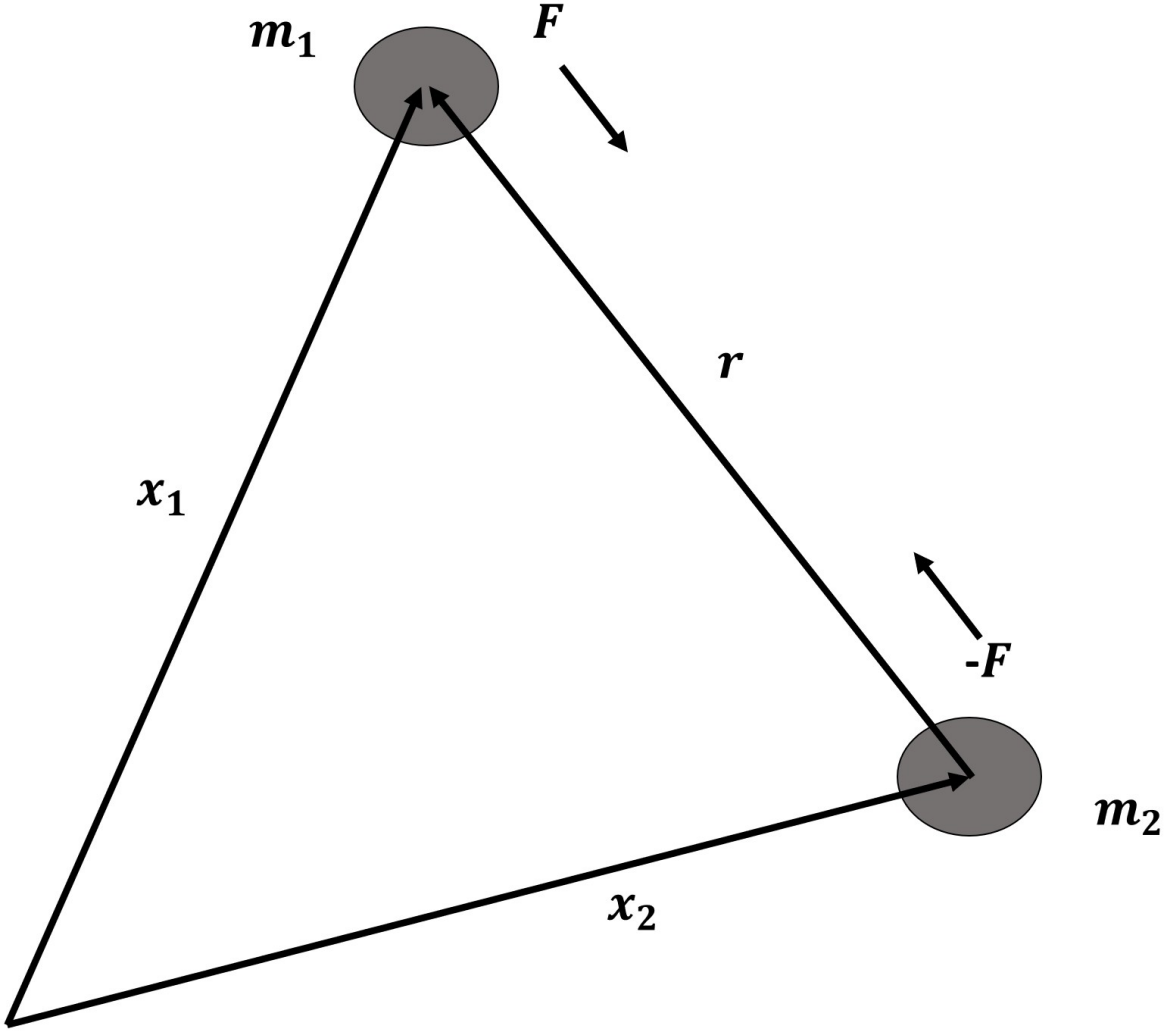
The two body problem.

*Example 6*: Newton’s equation of the two body motion problem refers to the movement of two particle interacting which each other due to gravitational pull, neglecting any third body the two do not collide with (see Fig 8)

In the current research, we consider the following formulation of the two body problem

$$\begin{array}{c} f_{1}^{\prime \prime }=-\frac{f_{1}}{\sqrt{f_{1}^{2}+f_{2}^{2}}},\;f_{2}^{\prime \prime }=-\frac{f_{2}}{\sqrt{f_{1}^{2}+f_{2}^{2}}},\;0\leq t\leq 10 \end{array}$$

with the initial conditions $f_{1}{|}_{0}=1\;\;f_{1}^{\prime }{|}_{0}=0,\;\;f_{2}{{|}_{0}=0,\;\;f_{2}^{\prime }|}_{0}=1$ and given analytical solution *f*_1_(*t*) = cos(*t*), *f*_2_(*t*) = sin(*t*). (Source: [36])

**Fig 9:**
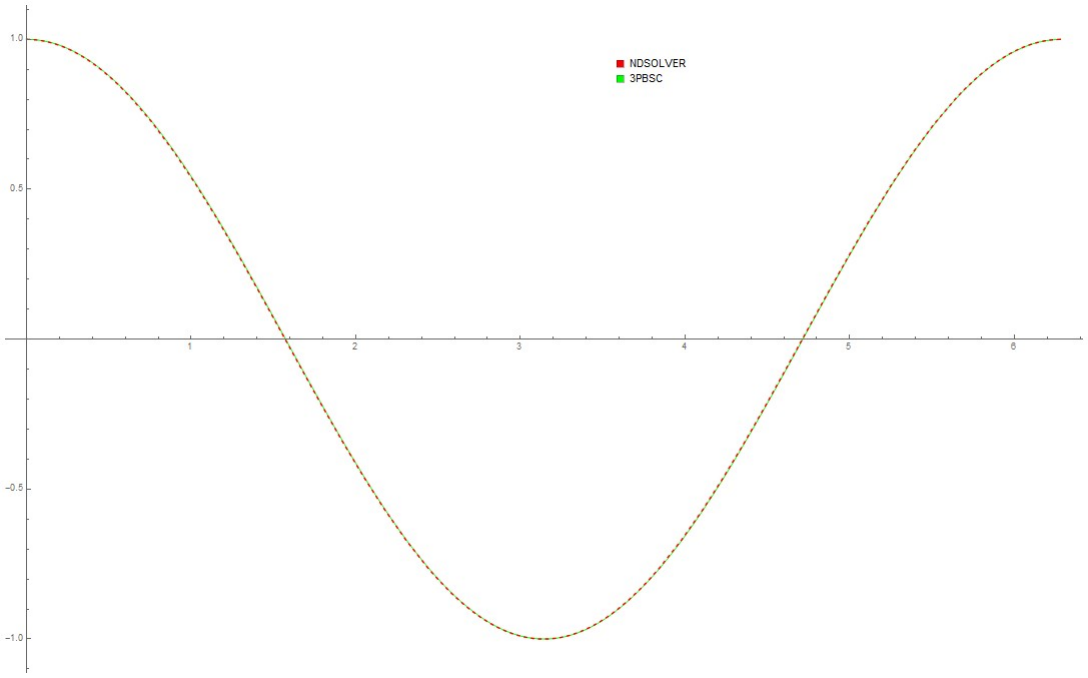
Comparison between NDSOLVER and 3PBCS for *f*_1_ of Example 6.

Data provided in Table 5 are numerical approximations for Example 6 using DI and 3PBCS method. Results exhibits that when using a larger step size, both methods are comparable but as smaller step size are used, 3PBCS begins showing an obvious advantage. Results produced in the table shows that the accuracy of 3PBCS improves at a rate of *H*^2^ compared to e the DI method which barely equals the current step size. To further validate the capability of the 3PBCS method, Figs 9 dan 10 show graphical approximation of *f*_1_ and *f*_2_ by NDSOLVER and 3PBCS methods. It is clear that both methods practically overlap each other, point by point. Fig 11 is presented to illustrate the orbit of the two body motion as approximated by 3PBCS. Example 6, with the current initial conditions presents a circular orbit (orbit 1), where as, by changing the initial conditions to $f_{1}{|}_{0}=0.4\;\;f_{1}^{\prime }{|}_{0}=0,\;\;f_{2}{{|}_{0}=0,\;\;f_{2}^{\prime }|}_{0}=2$, gives Example 6 a Kepler-like solution which produces an elliptic orbit (orbit 2) and can be verified from the work of Shampine and Gordon [37].

**Fig 10:**
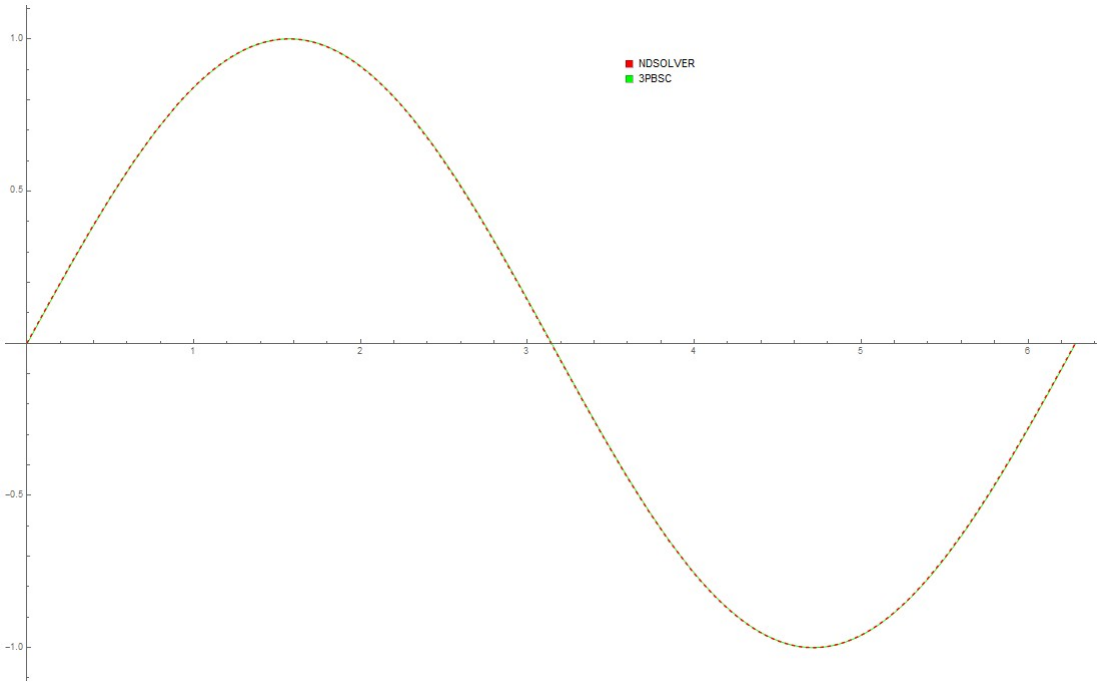
Comparison between NDSOLVER and 3PBCS for *f*_2_ of Example 6.

**Fig 11:**
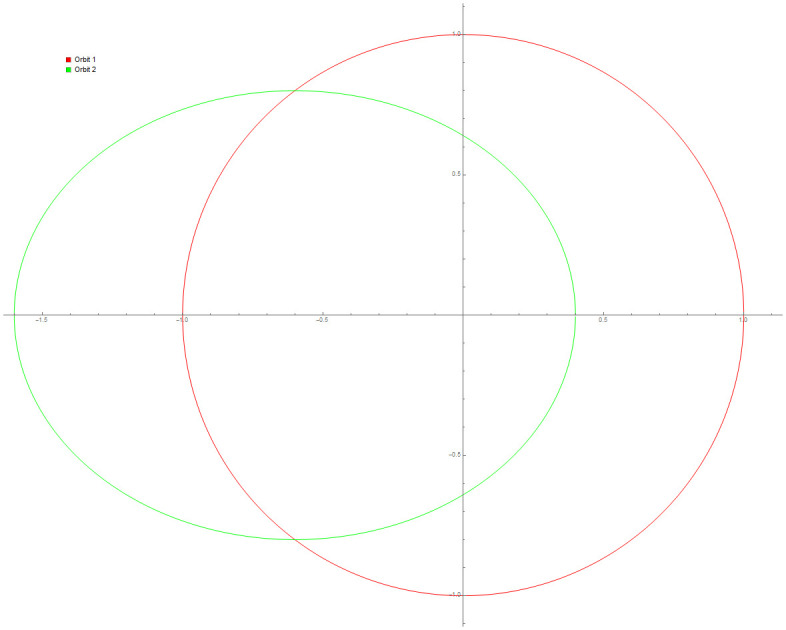
Orbit for Example 6 with different initial conditions.

**Table 5:** Comparisan of accuracy and total step of 5 different step size for Example 6.

H	Example 6			
	Method	TSteps	ErrEQ1	ErrEQ2
10^−1^	DI	100	7.86394e−02	9.00026e−02
	**3PBCS**	**34**	**2.37337e−02**	**2.11901e−02**
10^−2^	DI	1000	8.02804e−03	9.24779e−03
	**3PBCS**	**334**	**3.11795e−04**	**3.22462e−04**
10^−3^	DI	10000	8.02694e−04	9.28815e−04
	**3PBCS**	**3334**	**3.20106e−06**	**3.33937e−06**
10^−4^	DI	100000	8.02843e−05	9.29075e−05
	**3PBCS**	**33334**	**3.20816e−08**	**3.34984e−08**
10^−5^	DI	1000000	8.02848e−06	9.29096e−06
	**3PBCS**	**333334**	**3.00597e−10**	**3.43977e−10**

**Fig 12:**
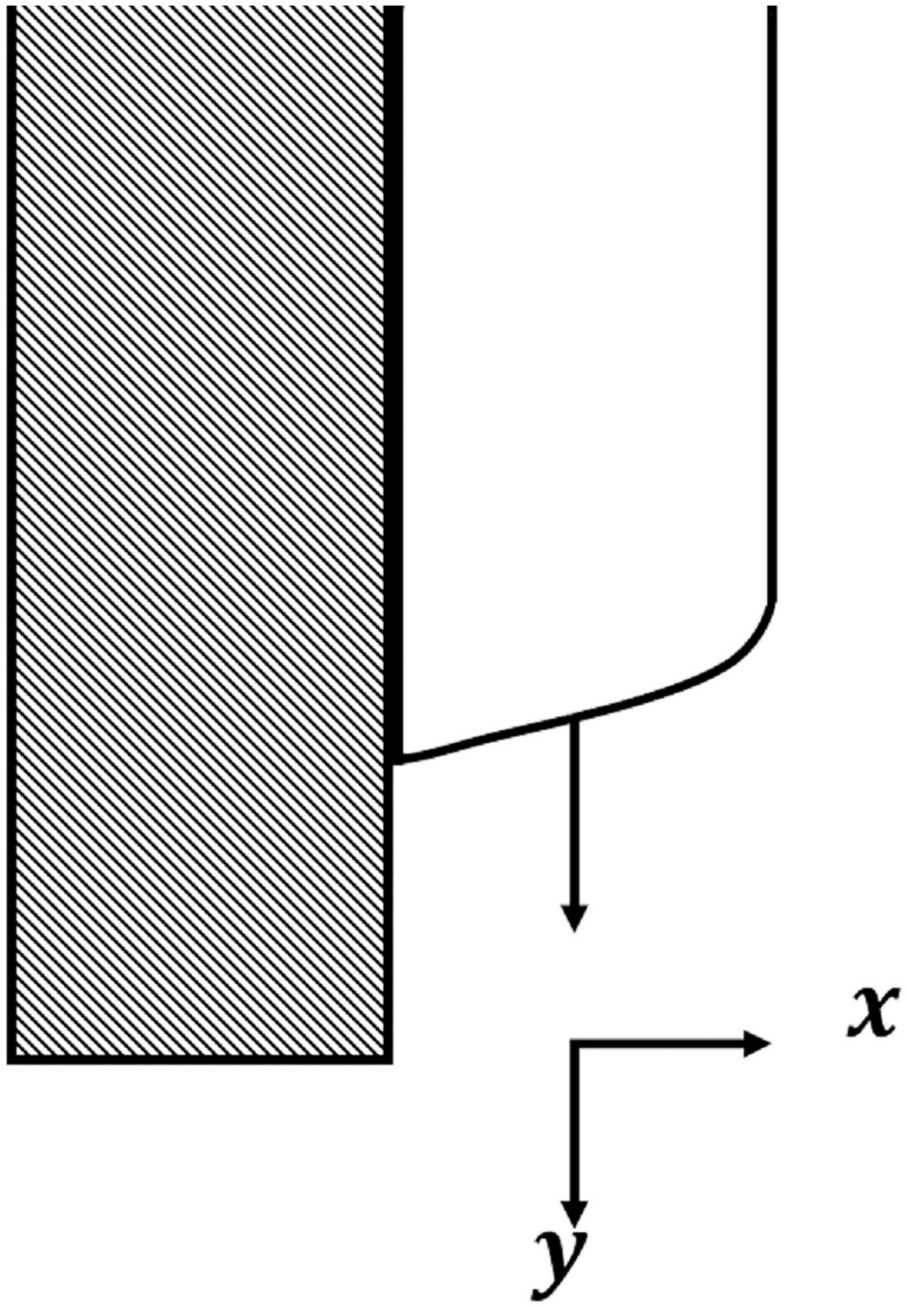
An idealized draining flow.

*Example 7*: Many problems in engineering and physics are based on the famous thin flow of liquid which is a third order ODEs with the ideal draining flow as shown in Fig 12.

These thin film flow problems can be found in various form like the the infamous drainage dry surface given by the function

$$\begin{array}{c} f^{\prime \prime \prime }=-1+f^{-2}. \end{array}$$

Consider a thin film pre-wetted surface with a remotely small thickness, *ω* > 0. This changes the function to

$$\begin{array}{c} f^{\prime \prime \prime }=-1+\left(1+\omega +\omega^{2}\right)f^{-2}-\left(\omega +\omega^{2}\right)f^{3}. \end{array}$$

Among the main concern is the tension surface effects when dealing with a flow of thin **film** of viscous fluid with free surface. As discussed in [38]

$$\begin{array}{c} f^{\prime \prime \prime }=f^{-k} \end{array}$$

with initial values *f*|_0_ = , *f*′|_0_ = 1, *f*′|_0_ = 1. (Source: [39]) exemplify the dynamic balance against the strength of a thin fluid layer, disregarding gravity.

**Fig 13:**
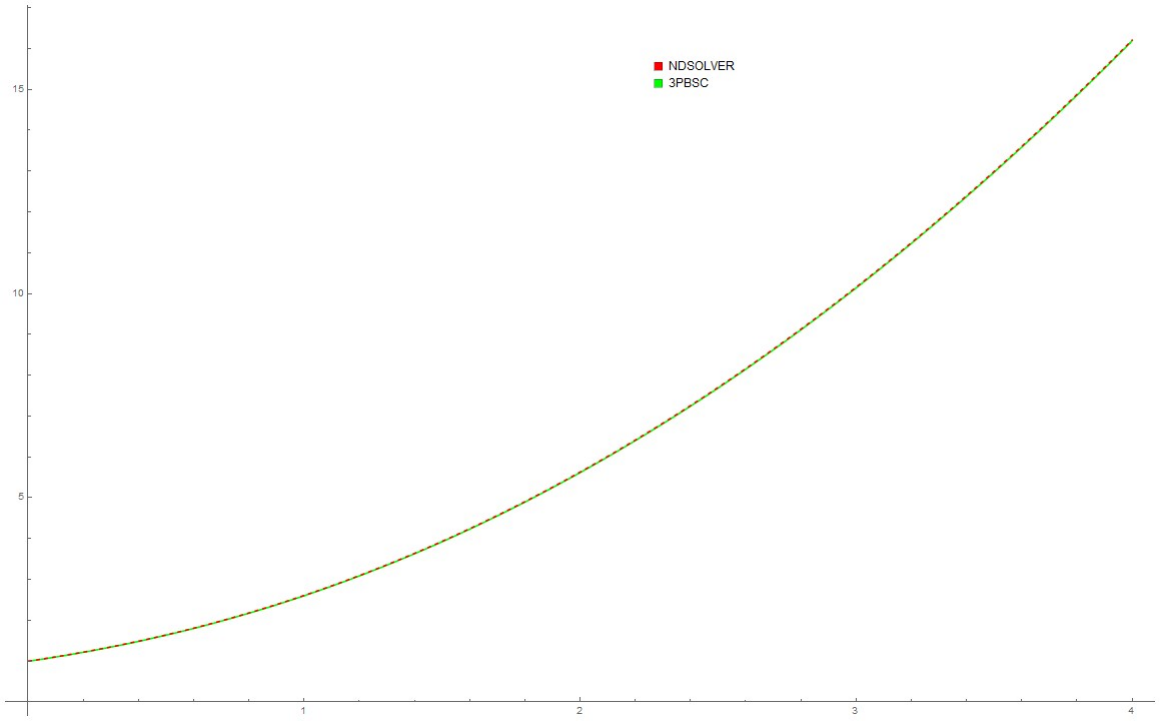
Comparison between NDSOLVER and 3PBCS for the solution of Example 6.

**Fig 14:**
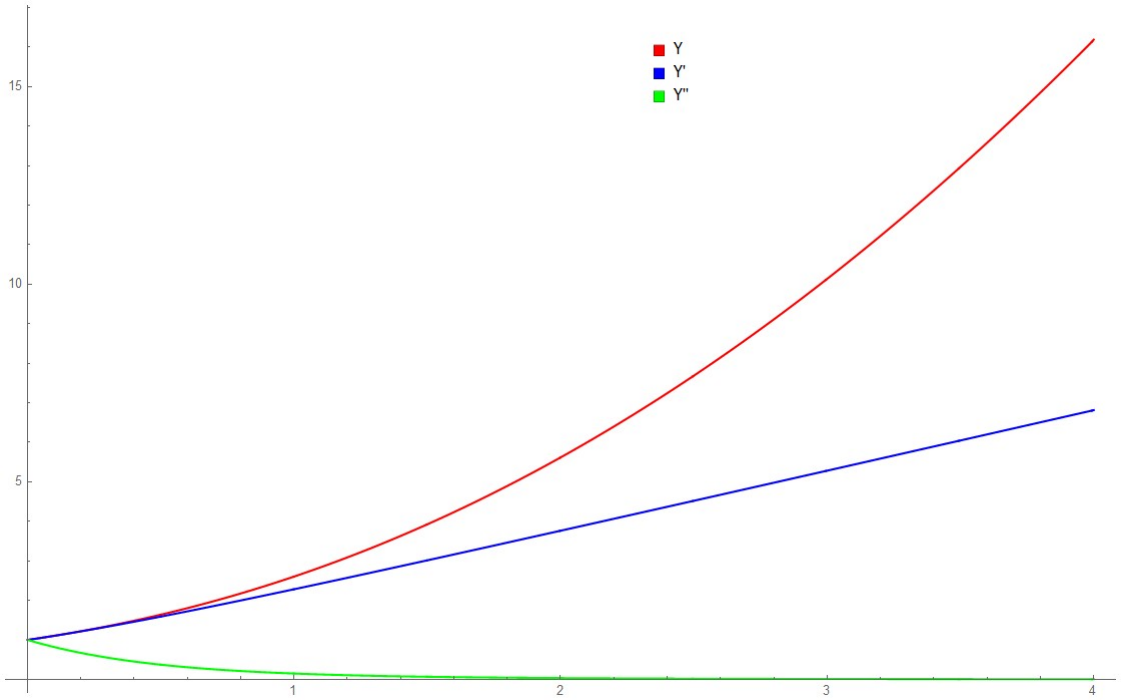
Numerical approximation of *f*, *f*′ and *f*″ by the 3PBCS method for Example 6.

Because the thin flow problem have no exact or analytical solution, the solution by 3PBCS is compared against the solution obtained by the DI. Table 6 presents the approximated solution obtained by 3PBCS method of *f*, *f*′ and *f*″ for the points 1.0, 1.5, 2.0, 2.5, 3.0, 3.5 and 4.0. As shown in Table 6, both methods are comparable with similar approximation up to at least 5 decimal points. Fig 13 compares solutions by NDSOLVER and 3PBCS. For the current example, 3PBCS method approximates the solution using a step size of *H* = 1 × 10^3^. Even at the current stepsize, the figure shows 3PBCS ability to match step by step solution of the NDSOLVER. Whereas, Fig 14 illustrates the difference of solution between *f*, *f*′ and *f*″.

**Table 6:** Numerical approximation by DI and 3PBCS method of points *f*, *f*′ and *f*″ for Example 7 given *k* = 2.

TOL	Method	*k* = 2		
		*f*	*f*′	*f*″
1.0	DI	2.60828e+00	2.28486e+00	1.46992e-01
	**3PBCS**	**2.60828e+00**	**2.28491e+00**	**1.46992e-01**
1.5	DI	3.93278e+00	3.01725e+00	6.46548e-02
	**3PBCS**	**3.93278e+00**	**3.01725e+00**	**6.46548e-02**
2.0	DI	5.62831e+00	3.76676e+00	3.15678e-02
	**3PBCS**	**5.62831e+00**	**3.76677e+00**	**3.15678e-02**
2.5	DI	7.70090e+00	4.52454e+00	1.68623e-02
	**3PBCS**	**7.70090e+00**	**4.52454e+00**	**1.68623e-02**
3.0	DI	1.01536e+01	5.28668e+00	9.69980e-03
	**3PBCS**	**1.01536e+01**	**5.28669e+00**	**9.69979e-03**
3.5	DI	1.29880e+01	6.05132e+00	5.92811e-03
	**3PBCS**	**1.29880e+01**	**6.05132e+00**	**5.92811e-03**
4.0	DI	1.62051e+01	6.81747e+00	3.80798e-03
	**3PBCS**	**1.62051e+01**	**6.81747e+00**	**3.80798e-03**

**Fig 15:**
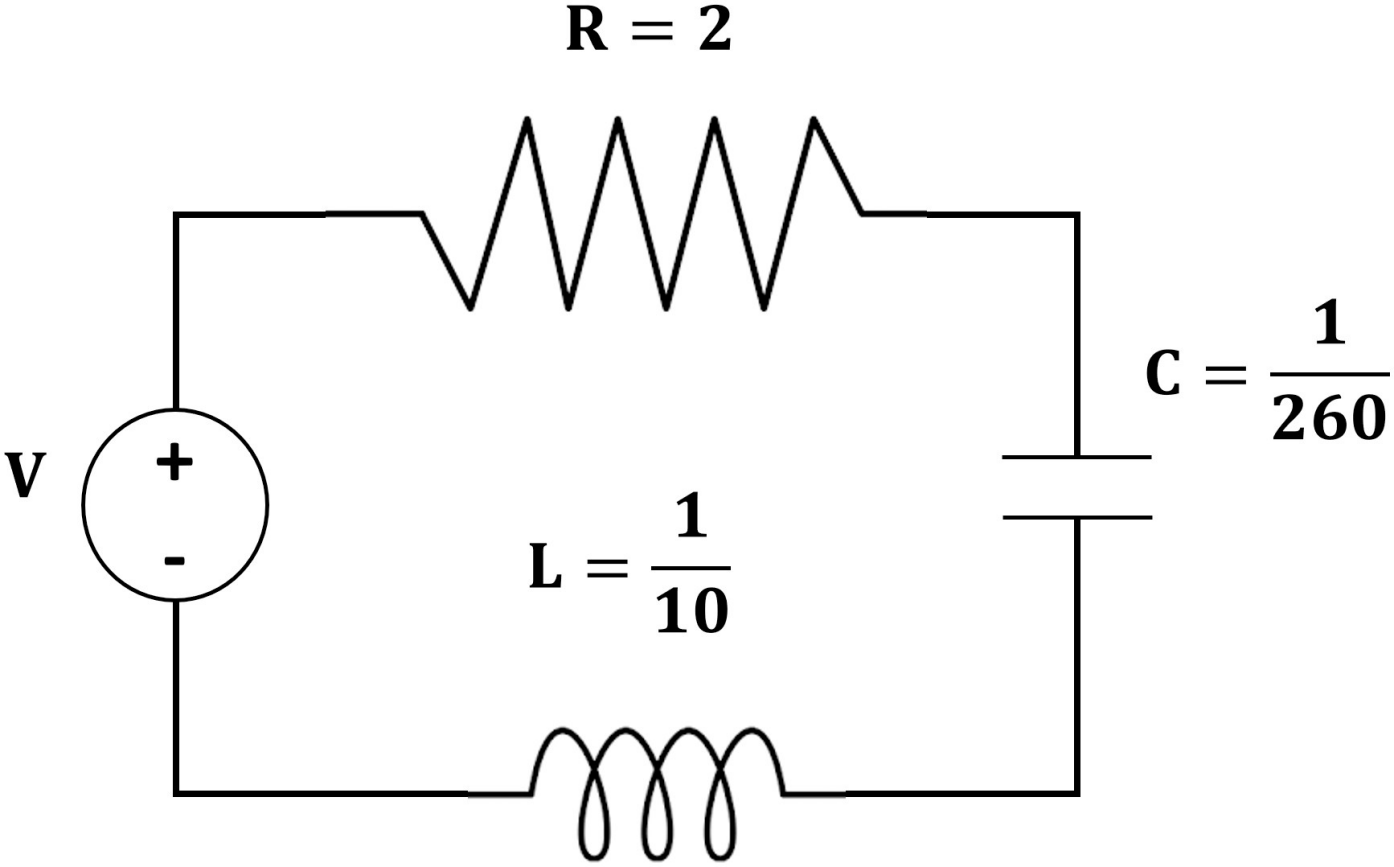
Second order RLC circuit.

*Example 8*: Consider the second order RLC circuit in Fig 15 below, with resistance of 2 ohm, capacitor at $\frac{1}{260}$ and inductor at 0.1 Henry and electrical force of 100 sin 60*tV*. The objective is to find the capacitor charge at any time, *t* > 0 given the initial current and initial charge of the capacitor are both zero. By Kirchhoff law, the following second order differential equation can be derived,

$$\begin{array}{c} f^{\prime \prime }=-20f^{\prime }-2600f+1000\text{sin}\left(60t\right) \end{array}$$

with the initial conditions *f*|_0_ = 0, *f*′|_0_ = 0 and given analytical solution $f\left(t\right)=\frac{6e^{-10t}}{61}\left(6\;\text{sin}\;\left(50t\right)+5\;\text{cos}\;\left(50t\right)\right)-\frac{5}{61}\left(5\;\text{sin}\;\left(60t\right)+6\;\text{cos}\;\left(60t\right)\right)$. (Source: [40])

**Fig 16:**
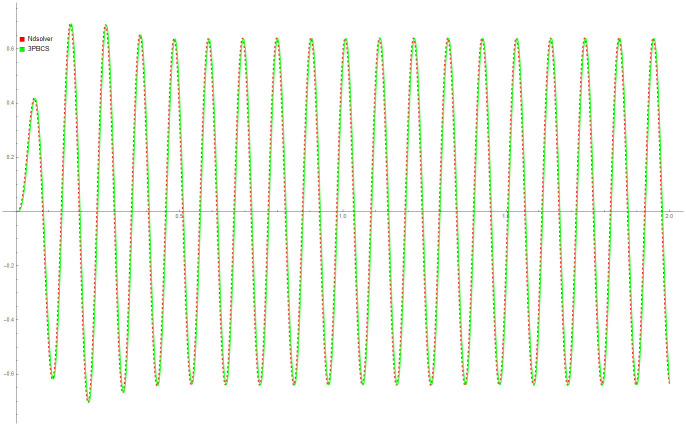
Comparison between NDSOLVER and 3PBCS for the solution of Example 7.

**Fig 17:**
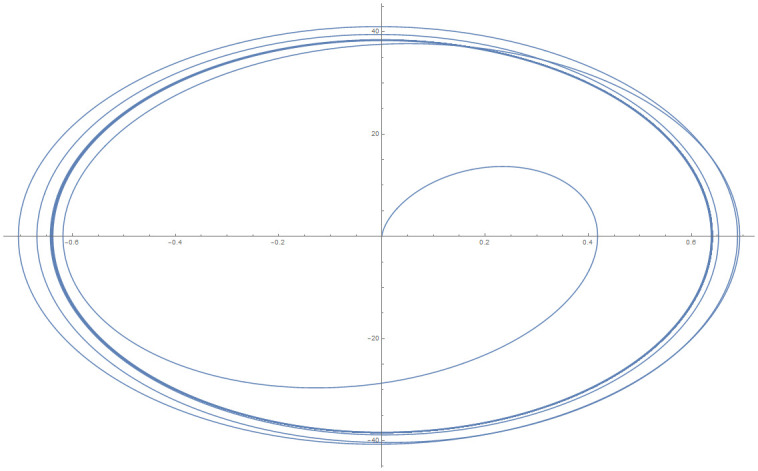
Parametric plot of *f* and *f*′ of 3PBCS method for Example 7.

The following Table 7 contains numerical results of 3PBCS method for example. The approximated values for end points (*T*_*n*_) 0.5, 1.0, 1.3 and 2.0 are provided with the corresponding exact solutions. The maximum error of all points, ErrMax is defined by *T*_0_ ≤ |*f*(*t*_*n*+*b*_) − *f*(*t*)| ≤ *T*_*n*_, *b* = 1, 2, 3 is also provided for a clearer view of 3PBCS abilities. As demonstrated in Table 6, 3PBCS is consistently accurate to the seventh decimal point when applying a stepsize of *H* = 1 × 10^−3^. Fig 16 is the approximate solution for *f*_*n*_ by NDSOLVER and 3PBCS. The graphical overlap shown in the figure exhibits the similar accuracy of both methods, whereas Fig 17 is parametric plot of Example 8 presented to express the level of difficulty of the problem.

**Table 7:** Comparison of approximated solution and exact solution for Example 8.

	Approximate *f*(*t*_*n*+3_)	Exact *f*_*n*_(*t*)	ErrMax *T*_0_ ≤ |*f*(*t*_*n*+*b*_) − *f*_*n*_(*t*)| ≤ *T*_*n*_
0.5	3.31828e-01	3.31828e-01	1.82051e-07
1	5.93337e-01	5.93337e-01	1.82051e-07
1.5	-1.46028e-01	-1.46028e-01	1.82051e-07
2	-6.38372e-01	-6.38372e-01	1.82051e-07

*Example 9*: The Van der Pol equation is an ODE that is derived from Rayleigh differential equation. In a Van der Pol equation,

$$\begin{array}{c} f^{\prime \prime }=2\xi \left(1-f^{2}\right)f^{\prime }-f, \end{array}$$

*f*(*t*) is defined as the displacement of the periodic solution and *f*|_0_ is the amplitude of the oscillation (Source: [41]). The classical nonlinear dynamics of a self sustained oscillation is commonly modeled when *ξ* > 0. Parameters used for this problem are *ξ* = 0.025 for 0 ≤ *t* ≤ 40 with initial conditions *f*|_0_ = 0, *f*′|_0_ = 0.5.

**Fig 18:**
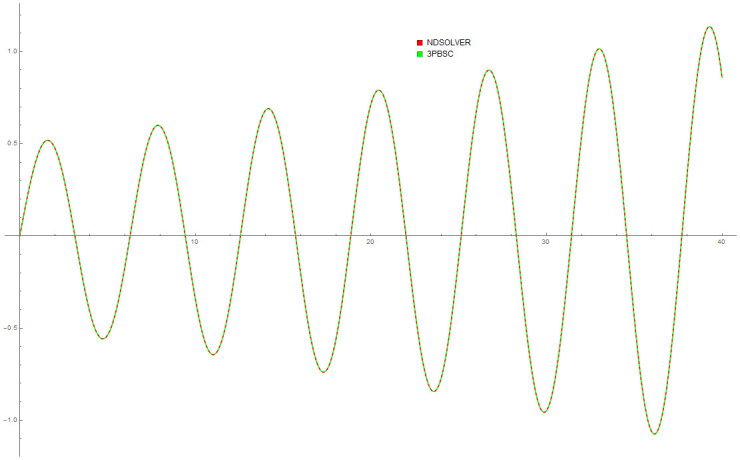
Numerical Comparison between NDSOLVER and 3PBCS.

**Fig 19:**
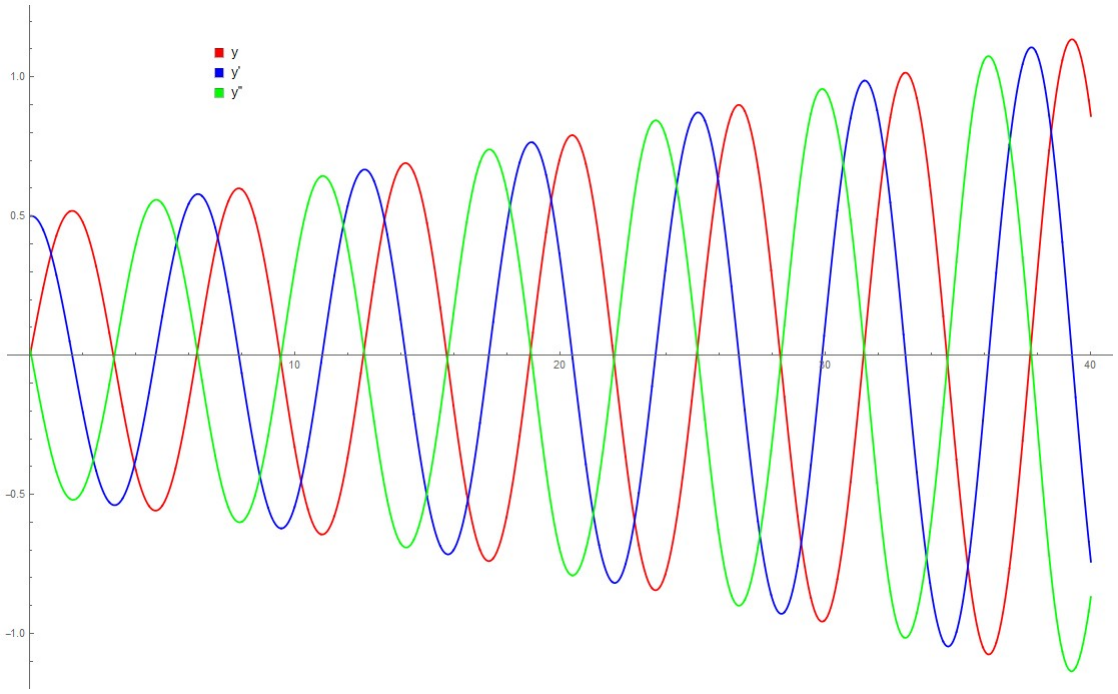
Aprroximation of *f*, *f*′ and *f*″ by 3PBCS method.

**Fig 20:**
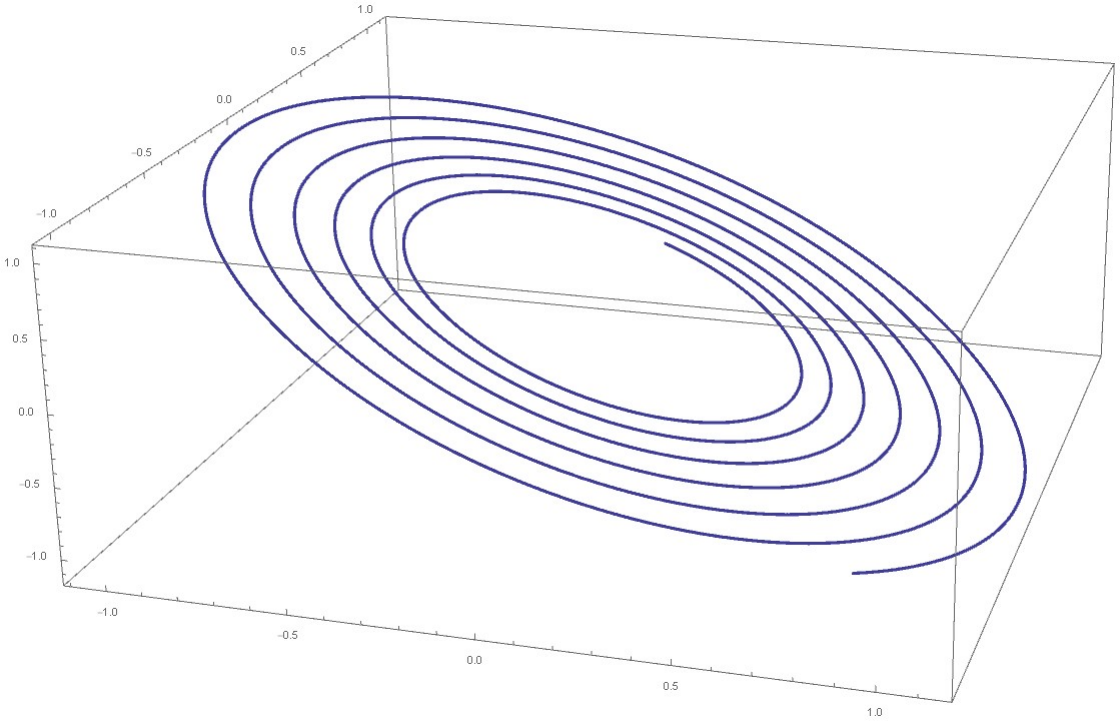
A 3D parametric plot of *f*, *f*′ and *f*″ approximated by 3PBCS method for Example 5.

Example 9 is originally a problem obtained from [42]. The Van Der Pol equation selected does not have any known analytical solution. Table 8 provides approximated values of *f*, *f*′ and *f*″ for 5 different end points by the DI and 3PBCS methods. Numerical approximation shows that both methods obtained similar values for *f*, *f*′ and *f*″ up to 1 × 10^−5^. Figs 18 and 19 illustrate the approximated solution, *f* by the 3PBCS method against NDSOLVER for the interval 0 ≤ *t* ≤ 40. Results in Fig 18 shows similar approximation for both 3PBCS and NDSOLVER where Fig 19 illustrates a graphical representation approximated values of *t* against *f*, *f*′ and *f*″. Fig 20 is to present a 3D illustration of *f*, *f*′ and *f*″ which corresponds to similar steps points by the 3PBCS method.

**Table 8:** Numerical result of 3PBCS method in constant order and step size mode for Example 8.

*T*_*n*_	Method	*f*	*f*′	*f*″
0.5	DI	2.4270367281e-001	4.5018020968e-001	-2.2152055722e-001
	**3PBCS**	**2.4270367388e-001**	**4.5018022195e-001**	**-2.2152055773e-001**
1.0	DI	4.3105071043e-001	2.8664149615e-001	-4.1938160270e-001
	**3PBCS**	**4.3105073032e-001**	**2.8664156431e-001**	**-4.1938162006e-001**
1.5	DI	5.1671714810e-001	4.8026186062e-002	-5.1495698025e-001
	**3PBCS**	**5.1671722009e-001**	**4.8026324566e-002**	**-5.1495704734e-001**
2.0	DI	4.7630950304e-001	-2.0708952836e-001	-4.8431485170e-001
	**3PBCS**	**4.7630965708e-001**	**-2.0708934721e-001**	**-4.8431499722e-001**
2.5	DI	3.1729761641e-001	-4.1655545180e-001	-3.3602849515e-001
	**3PBCS**	**3.1729786006e-001**	**-4.1655528657e-001**	**-3.3602872814e-001**
5.0	DI	-5.3857425618e-001	1.4704130967e-001	5.4379376516e-001
	**3PBCS**	**-5.3857445794e-001**	**1.4704079637e-001**	**5.4379394710e-001**

## Conclusion

Results provided in the current work validate that 3PBCS is a viable numerical method for solving ODEs. The three-point element of the method allows for lesser computational cost and provides better efficiency compared to its rival counterparts. By simply using a constant step size algorithm decreases computational cost and increases efficiency. Tables 1, 3 and 5 validate the convergence of the method. When a smaller *H* is selected, the method becomes more accurate and proven to provide a consistent set of accuracy for every point in the interval as indicated in Tables 4 and 7. For future works, the 3PBCS can be fitted with a variable order step size and parallel computing algorithm. The effectiveness of a variable order step size algorithm depends on the selection of a suitable order step size criterion. Authors a currently refining appropriate conditions to provide a better approximation for a variable order step size algorithm.
